# In Vitro Screening for Anti-Acetylcholinesterase and Antioxidant Activities of *Hottonia palustris* L. Extracts and Their Unusual Flavonoids

**DOI:** 10.3390/molecules27228034

**Published:** 2022-11-19

**Authors:** Jakub W. Strawa, Katarzyna Jakimiuk, Zuzanna Kita, Michał Tomczyk

**Affiliations:** 1 Department of Pharmacognosy, Faculty of Pharmacy with the Division of Laboratory Medicine, Medical University of Białystok, ul. Mickiewicza 2a, 15-230 Białystok, Poland; 2 Students’ Scientific Association, Department of Pharmacognosy, Faculty of Pharmacy, Medical University of Bialystok, ul. Mickiewicza 2a, 15-230 Białystok, Poland

**Keywords:** *Hottonia palustris*, Primulaceae, flavonoids, LC-HRMS, antioxidant activity, acetylcholinesterase inhibition

## Abstract

*Hottonia palustris* L. is from the genus *Hottonia* (Primulaceae), and the understanding of its phytochemical and pharmacological properties is limited. In this study, the use of chromatographic techniques led to the isolation of a further eleven compounds, including three new flavonoids: 2′,5-dihydroxyflavone 2′-*O*-*β*-glucopyranoside, 5,6-dihydroxyflavone 6-*O*-(6”-*O*-glucopyranosyl)-*β*-glucopyranoside (hottonioside A), and 4′,5,7-trihydroxyflavone 7-*O*-(2”-*O*-*β*-glucuronide)-*β*-glucopyranoside. Their structures were determined using extensive 1D and 2D NMR data and mass spectrometry (HRMS). The qualitative assessment of the chemical composition of the investigated extracts and fractions was performed using the LC-HRMS technique. Furthermore, the antioxidant potential of extracts, fractions, and compounds and their ability to inhibit acetylcholinesterase were also evaluated. Thus, we may conclude that the observed biological effects are the result of the presence of many biologically active compounds, of which dibenzoylmethane is the most active. Therefore, *H. palustris* is a source of substances with desirable properties in the prevention and treatment of neurodegenerative diseases.

## 1. Introduction

The genus *Hottonia* L. (Primulaceae) is represented by two confirmed flora species, *H*. *inflata* Elliott and *H*. *palustris* L., as well as one of ambiguous status, *H*. *serrata* Willd. *H*. *inflata* is a species that is native to the flora of North America. It is commonly referred to as the American featherfoil [1,2]. Previous studies indicate that the use of this species in folk medicine is helpful for individuals with heart disease [3]. The Eurasia region is the habitat of the second species, *H*. *palustris* [4,5,6]. Previous studies of its lipophilic extracts allowed the isolation of new and biologically active methoxyflavones [7], while an analysis of its alcoholic and hydroalcoholic extracts led to compound isolations [8]. Considering the medicinal potential of *H*. *palustris*, this study aimed to isolate further novel bioactive flavone and flavonol derivatives. A further nineteen compounds were isolated and characterized by spectroscopic and spectrometric analyses, five of which are described detail for the first time. Moreover, detailed qualitative and quantitative analyses of the extracts were carried out, and an evaluation of the antioxidant potential of the extracts and compounds was completed. Finally, we addressed the growing interest in the search for new, as well as known, effective substances to inhibit the neurodegenerative processes underlying the etiology of Alzheimer’s disease [9,10,11,12] and carried out an evaluation of the inhibitory effect on acetylcholinesterase.

## 2. Results and Discussion

**Figure 1:**
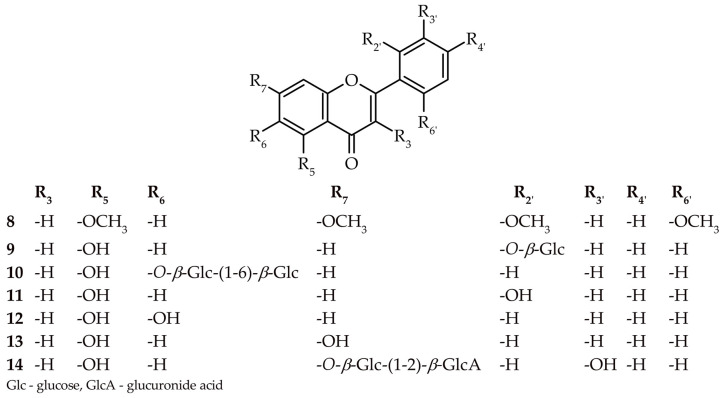
Chemical structures of isolated flavones (**8–14**).

**Figure 2:**
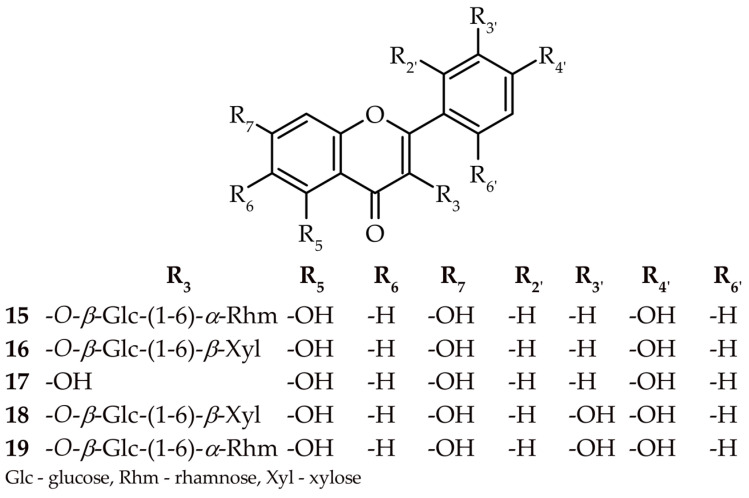
Chemical structures of the isolated flavonols (**15–19**).

A detailed study of chloroform extract, as well as ethyl acetate and n-butanol fractions from the *H. palustris* herb, led to the isolation of eleven compounds. These were identified as 5,6,2′,6′-tetramethoxyflavone (zapotin) (**8**), 5,2′-dihydroxyflavone 2′-*O*-β-glucopyranoside (**9**) 5,6-dihydroxyflavone 6-*O*-(6″-*O*-glucopyranosyl)-β-glucopyranoside (hottonioside A) (**10**), 5,2′-dihydroxyflavone (**11**), 5,6-dihydroxyflavone (**12**), 5,7-dihydroxyflavone (chrysin) (**13**), apigenin 7-*O*-(2″-*O*-β-glucuronide)-β-glucopyranoside (**14**), kaempferol 3-*O*-(6″-*O*-α-rhamnopyranosyl)-β-glucopyranoside (nicotiflorin) (**15**), kaempferol-3-*O*-(6″-*O*-β-xylopyranosyl)-β-glucopyranoside (**16**), kaempferol (**17**), quercetin 3-*O*-(6″-*O*-β-xylopyranosyl)-β-glucopyranoside (**18**), and quercetin 3-*O*-(6″-*O*-α-rhamnopyranosyl)-β-glucopyranoside (rutin) (**19**) (see Figure 1 and Figure 2). Moreover, evidence of the structures of compounds **9, 10**, **14** was discovered for the first time. Notably, compounds **16** and **18** were characterized in meticulous detail for the first time.

### 2.1. Determination of Isolated Compounds ***8–19***

The chemical structures of compounds **8–19** were elucidated by their UV, HRMS spectrum, similarity with the retention times of commercial standards, and ^1^H and ^13^C NMR data, as well as through 2D NMR correlation experiments. The ultraviolet spectra of compounds **15–19** showed similar absorption bands at maxima of 253–268 and 350–368 nm, indicating a C3-O-X-free or substituted flavonol backbone. The values of the band maxima in the others suggest a lack of substitution at C-3, whereas differences in the values demonstrate the varying degree of oxidation of the A and B rings of flavone [13]. The analysis of the HRMS spectra of compounds **9**, **10**, **14**, **15–16**, and **18–19** showed the loss of neutral fragments, such as M-132, M-146, M-162, and M-174 *m*/*z*, representing pentose, deoxy-hexose, hexose, and uronic acid, which indicates the glycosidic nature of these compounds. More detailed information was provided by an in-depth analysis of correlated spectral and spectroscopic data of selected compounds.

#### 2.1.1. 5,2′-Dihydroxyflavone 2′-*O*-*β*-glucopyranoside (**9**)

**Figure 3:**
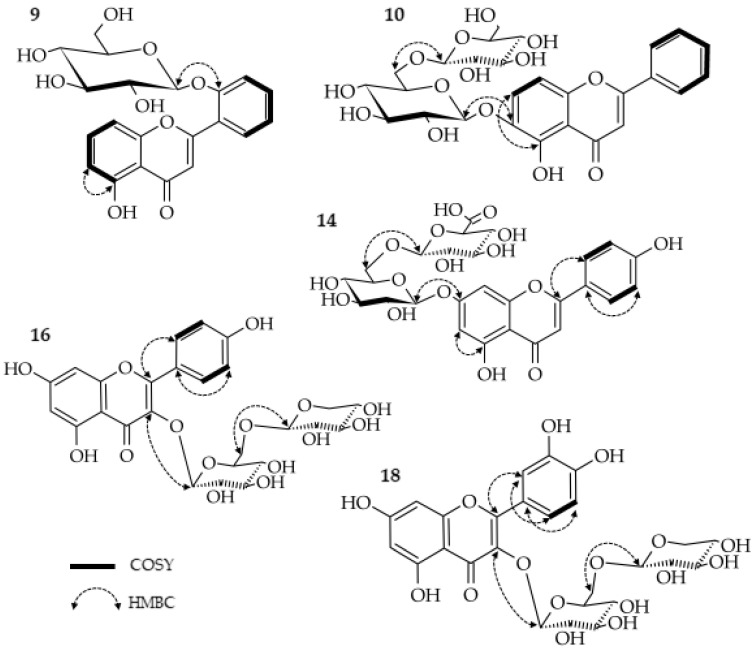
Relevant COSY and HMBC correlations occurring in the spectra of compounds **9**, **10**, **14**, **16**, and **18** isolated from *H*. *palustris*.

Compound **9** was obtained as a white, amorphous powder. Its molecular formula was determined to be C_21_H_20_O_9_ by ^13^C NMR, and the pseudomolecular ion *m/z* 417.1187 [M+H]^+^ was recorded (see Figures S1 and S2). The ^1^H NMR displayed typical signals for flavones, such as olefinic δ_H_ = 7.16, which corresponded to a C-3 value of δ_C_ = 110.78 [14], as well as a recorded UV spectrum (see Figures S3 and S4). The ^1^H NMR spectrum showed a downfield signal at δ_H_ = 12.71, which could be attributed to a C-5 hydroxyl group (see Figure S3). The correlation of protons in COSY confirmed substitution at the C-5 positions in ring A, as well as at C-2′ in ring B (see Figure S5). The assignment of protons attached directly to carbons was carried out based on HSQC (see Figure S6). Furthermore, an anomeric proton signal at δ_H_ = 5.13, (d, *J* = 7.03 Hz) suggested the presence of a sugar residue with a *β*-configuration. This was confirmed to be glucose when **9** was subjected to acid hydrolysis. The HMBC correlation supported the presence of a glycosidic bond by correlating the anomeric proton with C-2′ (δ_C_ = 155.63) (see Figure 3 and Figure S7). On the basis of the foregoing studies, the structure of **9** was established as 5,2′ -dihydroxyflavone 2′-*O*-*β*-glucopyranoside. To the best of the authors’ knowledge, the structure is new and has not yet been characterized in the literature. The NMR data are provided in Table 1.

#### 2.1.2. 5,6-Dihydroxyflavone 6-*O*-(6″-*O*-*β*-glucopyranosyl)-*β*-glucopyranoside (hottonioside A) (**10**)

Compound **10**, obtained as a yellow, amorphous powder, gave HRMS and ^13^C NMR spectra indicating the formula C_27_H_30_O_14_ (see Figures S8 and S9). The mentioned compound exhibited ultraviolet absorption at 279, 310*sh* (shift), and 345 nm (see Figure S10) and showed a pseudo-molecular ion at *m*/*z* 579 [M+H]^+^ and ions at *m*/*z* 417 [M+H-162]^+^ and *m*/*z* 255 [M+H-162-162]^+^, which suggests the existence of two hexoses. These conclusions were proved by an investigation of the hydrolysis products. The presence of glucose and aglycon corresponding to compound **11** was confirmed. The ^1^H NMR displayed typical signals for flavones, such as olefinic δ_H_ = 7.03, which corresponded to a C-3 value of δ_C_ = 104.97 [14]. It is noteworthy that the proton spectrum lacks a signal for H-6, and the signals of H-7 and H-8 are shifted downfield. However, a hydroxyl group connected to C-5 is present (δ_H_ = 12.73, s) (see Figure S11). The COSY analysis confirmed a lack of substitution in the B ring (see Figure S12). Directly attached protons were assigned using HMQC (see Figure S13). The glycosylation link of both glucosyl units was determined using an HMBC experiment, which showed a cross-peak between the H-1″ doublet (δ_H_ = 4.92, *J* = 6.53 Hz) and the C-6 carbon (δ_C_ = 140.59). The second unit was based on a cross-peak between the H-1′″ doublet (δ_H_ = 4.2, *J* = 7.78 Hz) and the C-6″ carbon (δ_C_ 68.76), forming a gentiobiose moiety (see Figure 3 and Figure S14). In summary, this precise analysis of the ultraviolet and mass spectra, as well as one and two-dimensional NMR data, provides sufficient evidence to establish compound **10** as 5,6-dihydroxyflavone 6-*O*-(6″-*O*-*β*-glucopyranosyl)-*β*-glucopyranoside or 5,6-dihydroxyflavone 6-*O*-gentobioside as a new natural product named hottonioside A. Detailed data from the NMR experiments are provided in Table 1.

#### 2.1.3. Apigenin 7-*O*-(2″-*O*-*β*-glucuronide)-*β*-glucopyranoside (**14**)

Compound **14** was obtained as a light-brown, amorphous powder. The HRMS showed an [M−H]^−^ pseudomolecular ion at *m*/*z* 607.13. The ^13^C NMR and HRMS data suggested a molecular formula of C_27_H_28_O_16_ (see Figures S15 and S16). The UV spectrum (270, 332 nm) indicates that the compound belongs to the flavone family (see Figure S17), while the MS data revealed the presence of a serially *O*-substituted aglycone *m*/*z* 271, [M+H-338]^+^ with a glycosyl moiety. The product of hydrolysis proved the presence of glucose and glucuronic acid, as well as apigenin. The ^1^H NMR and COSY spectra exhibited signals characteristic for apigenin, as well as signals of two anomeric protons (δ_H_ = 5.2, d, *J* = 7.03 Hz; δ_H_ 4.55, d, *J* = 7.78 Hz) (see Figure S18 and S19)*.* The presence of the second carbonyl group confirmed the presence of a uronic acid moiety by a signal at 170.67 ppm in the ^13^C NMR spectrum (see Figure S18). Directly attached protons were assigned using HMQC (see Figure S20). The glycosylation position was determined by the HMBC experiment, which revealed a glucose cross-peak between the H-1″ doublet (δ_H_ = 5.2) and the C-7 carbon (δ_C_ = 162.76). The glucuronic acid linkage was established based on the cross-peak between the H-1′″ doublet (δ_H_ = 4.55) and the C-2″ carbon (δ_C_ = 82.74) (see Figure 3 and Figure S21). In summary, the obtained data provide sufficient evidence to establish compound **14** as the first reported apigenin 7-*O*-(2″-*O*-*β*-glucuronide)-*β*-glucopyranoside. Detailed data from the NMR experiments are given in Table 1.

#### 2.1.4. Kaempferol 3-*O*-(6″-*O*-*β*-xylopyranosyl)-*β*-glucopyranoside (**16**)

Compound **16** was obtained as a yellow, amorphous powder. The HRMS showed an [M−H]^−^ predominant ion at *m*/*z* 579.1358. The HRMS and ^13^C NMR data suggested a molecular formula of C_26_H_28_O_15_ with a mass difference of 0.6 ppm (see Figures S22 and S23). The UV spectrum (268, 351 nm) indicates that this compound belongs to the flavonol family (see Figure S24). Examination of the hydrolytic degradation products yielded glucose and xylose moieties, as well as kaempferol as aglycone. The proton spectrum confirmed signals corresponding to the suspected flavonol [13] and two doublets from the anomeric protons of the attached sugars (δ_H_ = 5.37,d, *J* = 7.53 Hz; δ_H_ = 4.00, d, *J* = 7.28 Hz). Finally, the value of the coupling constant indicated that the glycosides were *β*-anomers (see Figure S25). The protons assigned using COSY and HMQC confirmed the above conclusions (see Figure 3, Figures S26 and S27). The glycosylation link of glucose was determined by an HMBC experiment, which showed a cross-peak between the H-1″ doublet (δ_H_ = 5.37) and the C-3 carbon (δ_C_ = 133.69). The attachment of xylose was determined by a cross-peak between the H-1″’ doublet (δ_H_ = 4.00) and the C-6″ carbon (δ_C_ = 68.35) (see Figure S28). After evaluation of the 1D and 2D NMR spectra, compound **16** was established as kaempferol 3-*O*-(6″-*O*-*β*-xylopyranosyl)-*β*-glucopyranoside. The molecule deduced from the data had already been discovered, but the erroneous [15] and incomplete data encouraged the current authors to characterize it accurately (Figures S22–S28 and Table 1).

#### 2.1.5. Quercetin 3-*O*-(6″-*O*-*β*-xylopyranosyl)-*β*-glucopyranoside (**18**)

Compound **18**, obtained as yellow, amorphous powder, was characterized by a UV spectrum that is typical for flavonols (258, 300*sh*, 359 nm) [13] (see Figure S29). The analysis of the ^13^C NMR and HRMS spectra permitted the determination of the molecular formula C_26_H_28_O_16_ (see Figures S30–S31). The proton spectrum confirmed the presence of signals that, according to the literature, correspond to quercetin [13]. In the spectrum, the two visible doublets in the low chemical field (δ_H_ = 5.37, d, J = 7.28 Hz; δ_H_ = 4.01, d, J = 7.28 Hz) suggested the presence of two sugar molecules linked by bonds in the β-configuration (see Figure S32). An analysis of the hydrolytic degradation products also confirmed the presence of quercetin, glucose, and xylose. Protons directly attached to the carbons were assigned by COSY and HMQC analyses (see Figures S33 and S34). The manner of attachment of sugars was carried out using the HMBC correlation. The cross-peak visible in the spectrum between the H-1″ doublet (δ_H_ = 5.37) and the C-3 carbon (δ_C_ = 133.69) points out the attachment of a glucose molecule. The binding of the xylosyl unit was based on the cross-peak between the H-1″ doublet (δ_H_ = 4.00) and the C-6′ carbon (δ_C_ = 68.35) (see Figure 3 and Figure S35). All data presented above indicate that compound **18** is quercetin 3-O-(6″-O-β-xylopyranosyl)-β-glucopyranoside.

### 2.2. Evaluation of H. palustris Metabolome

#### 2.2.1. Qualitative LC-PDA-HRMS Analysis of *H. palustris* Extracts and Fractions

To understand the metabolome of *H*. *palustris*, extracts with a wide range of polarities were analyzed, as well as fractions characterized by the selective accumulation of specific groups of compounds. The LC-PDA-HRMS analysis revealed the presence of 31 compounds attributed to two main groups: flavonoids and triterpenes. Extremely non-polar extracts (**HP6–HP8**) were characterized by the presence of mainly methoxylated 5-hydroxyflavone derivatives (**P22–23**, **P25–26**, **P28**), monohydroxyflavone (**P27**), and dihydroxyflavones (**P18**, **P20**, **P24**), particularly in sugar combinations (**P12**). Extracts with moderately polar and polar properties were rich sources of flavones (**P2**, **P9–10**) and flavonol (**P1**, **P3–8**) diglycosides with a small representation of their free forms (**P11**, **P13–14**). In addition, a diketone representative (**P29**), dibenzoylmethane, was found, as well as significant compounds with high retention to a non-polar phase with unassigned identity (**P30–31**). Triterpene compounds (**P15–17**, **P19**), which are characteristic chemophenetic markers of the Primulaceae family, were also detected through the use of a mass spectrometer. Detailed chromatographic data are shown in Table 2 and Figures S37–S44 in the Supplementary Material.

#### 2.2.2. Quantitative Characteristics (TPC, TFC, TPAC, TTC) of *H. palustris* Extracts and Fractions

Except for compounds detected using the LC-MS method, other phytoconstituents and their synergic actions may be responsible for the biological activity of extracts and fractions from *H. palustris*. The total contents of phenolic (TPC), flavonoid (TFC), phenolic acids (TPAC), and tannins (TTC) are presented in Table 3. The total phenolic content of *H. palustris* extracts and fractions (**HP1–HP8**) varied from 12.44 (**HP3**) to 67.81 (**HP4**) mg Peq/g, while the TFC ranged from 1.62 (**HP3**) to 9.23 (**HP6**) mg Leq/g. Based on these values, **HP6** (3.03 mg CAeq/g) and **HP7** (3.48 mg CAeq/g) contained the highest total phenolic acid contents, while the total tannin content was elevated in **HP5** (5.40 mg Peq/g).

### 2.3. Assessment of the Antioxidant and Acetylcholinesterase Inhibiting Activities

The need to assess the antioxidant potential of natural products is well documented. Such measurements were conducted to moderate the effects of oxidative stress in the surrounding healthy cells, leading to the amplification of pathological processes. Furthermore, the protective roles of chelator agents against the development of neurodegenerative disease have been reported previously [16,17]. The antioxidant capacity of the samples was evaluated via four different methods, including radical scavenging (DPPH, ABTS), reducing power (CUPRAC), and metal chelating (FRAP) assays (Table 4 and Table 5).

Based on the scores outlined in Table 4, **HP3** and **HP8** were shown to possess the lowest antioxidant activity with all tested methods. Among the tested compounds, **12**, **18**, and **19** were found to be the leading antiradical agents, while **1–5** and **7** exhibited the lowest antioxidant activity. It is worth noticing that compounds **1–7** were included in this study to assess qualitative content and anti-acetylcholinesterase or antioxidant potentials [7].

These results can be understood as demonstrating the compounds with high total phenolic/flavonoid contents in extracts and fractions. Moreover, the limitation of the antioxidant potential of all tested compounds can be related to the methylation of the hydroxyl group in the flavonoid B-ring, as shown in compounds **1–5** and **7**. These conclusions are supported by previously reported research and available literature data [18,19].

In recent times, the involvement of enzyme inhibition in human diseases has been considered one of the most accessible therapeutic strategies in illness therapy. This aspect makes acetylcholinesterase inhibitors the principal class of drugs used for the treatment of Alzheimer’s disease (AD), among which galantamine (IC_50_ = 91.23 ± 0.52 µg/mL) is the only naturally occurring substance. At this point, there is growing interest in the use of plant-based natural enzyme inhibitors to avoid the side effects of available treatments [20]. The acetylcholinesterase (AChE) inhibitory activity of *H. palustris* and its main metabolites is reported in Table 4 and Table 5. The extracts and fractions were graded from the strongest to the weakest, as follows: **HP2** > **HP8** > **HP7** > **HP1** > **HP6** > **HP4**. **HP3** and **HP5** showed no significant inhibitory activity, probably because they have the lowest levels of methoxylated flavonoids. All nineteen compounds exhibited restrained anti-acetylcholinesterase activity. The greatest activity of all compounds isolated from *H. palustris* was observed in compound **1** (IC_50_ = 144.83 ± 1.30 µM). In the available literature data, the determination of the structure–activity relationship for flavonoids is complicated due to their diverse chemical structures. For example, some studies imply that methylation of the hydroxyl group at C4′ and the presence of a 7-*O*-sugar moiety are crucial for AChE inhibition [21,22]. The demonstrated strong biological activity of **HP1**-**HP7** correlates with the activity of individual compounds (**1–19**) present in the analyzed fractions and extracts. The identification of apigenin 7-*O*-(2″-*O*-β-glucuronide)-β-glucopyranoside apigenin (**14**) showed its dominant content in **HP3** and **HP5** fractions. In the **HP2** fraction, the dominant compounds are rutinosides of quercetin (**19**) and kaempferol (**15**). In addition, kaempferol (**17**) presents in many fractions and extracts and cannot be overlooked for the demonstrated biological activity. In the case of the **HP4** fraction, the effect may also depend on the presence of kaempferol 3-*O*-(6’-*O*-β-xylopyranosyl)-β-glucopyranoside (**16**) and the dominant nicotiflorin (**15**). For **HP1** and **HP8**, the effect is probably the result of the entire metabolite complex, although **HP1** is characterized by compounds such as kaempferol (**17**), zapotin (**8**), 5-hydroxy-2’-methoxyflavone (**3**), and the diketone-1,3-diphenylpropan- 1,3-dione (**1**). The **HP6** and **HP7** extracts can be characterized as a mixture of methoxyflavones, where zapotin (**8**), 5-hydroxy-2’-methoxyflavone (**3**), and 1,3-diphenylpropane-1,3-dione (**1**) can be considered the most biologically active compounds.

## 3. Materials and Methods

### 3.1. Chemicals and General Experimental Procedures

Comprehensive descriptions of the reagents used in the extraction, isolation, and identification processes, as well as in the evaluation of the antioxidant potential and acetylcholinesterase inhibition, are included in the Supplementary Materials. Notably, the aglycone standards used for the hydrolytic decomposition product and LC-MS analysis, apigenin, kaempferol, and quercetin (purity > 96%), were isolated from the leaves and flowers of *Arctium tomentosum* Mill. (Asteraceae) [23]. Compounds: 1,3-diphenylpropane-1,3-dione (**1**), 5-hydroxyflavone (**2**), 5-hydroxy-2′-methoxyflavone (**3**), 5-hydroxy-2’,6’-dimethoxyflavone (**4**), 5-hydroxy-2’,3’,6’-trimethoxyflavone (**5**), 2’,5-dihydroxy-6-methoxyflavone (**6**), and 5,6′-dihydroxy-2′,3′-dimethoxyflavone (**7**) used for qualitative HPLC analysis and evaluation of acetylcholinesterase inhibition have been isolated from the petrol extract of *H. palustris* herb (purity > 98%, HPLC) [7].

### 3.2. Plant Materials

According to our previous studies [7], the plant material consisted of leafy stems of *Hottonia palustris* L. (**HP**) (Primulaceae), collected without crawling roots or flowers (Podlasie, Poland; GPS: 53°17′13.2″ N, 22°53′42.0″ E). The species was authenticated based on the literature [8]. A voucher specimen (No. HP-17040) was deposited in the plant collection of the Department of Pharmacognosy, Medical University of Białystok, Poland.

### 3.3. Preparation of Extracts and Isolation Procedure

#### 3.3.1. Extraction

After purification in a Soxhlet apparatus and the collection of petrol (**HP6**) and chloroform (**HP7**) extracts and the chloroform fraction (**HP8**) [7], air-dried parts of *H. palustris* were extracted with solvents of increasing polarity. The freeze-dried part of the aqueous residue was dissolved and then fractionated by liquid–liquid extraction with ethyl acetate (75 × 250 mL) and butan-1-ol (110 × 250 mL). The combined organic layers were evaporated to dryness to yield 10.5 g of ethyl acetate residue (**HP4**), 76 g of butan-1-ol residue (**HP5**), and aqueous residue. Afterwards, to isolate the phenolics, the **HP4–HP6** residues were purified. Ultrasound-assisted extraction was used to prepare crude extracts: methanolic (**HP1**), 50% methanolic (**HP2**), and aqueous (**HP3**). Powdered plant parts (15 g) were etched with a proper solvent five times (temperature below 40 °C). Then, the combined portion of the extract was centrifuged, filtered, and evaporated to dryness under a vacuum. Finally, residues were suspended in water and then frozen and lyophilized.

#### 3.3.2. Isolation Using the MPLC and HPLC Techniques

**Figure 4:**
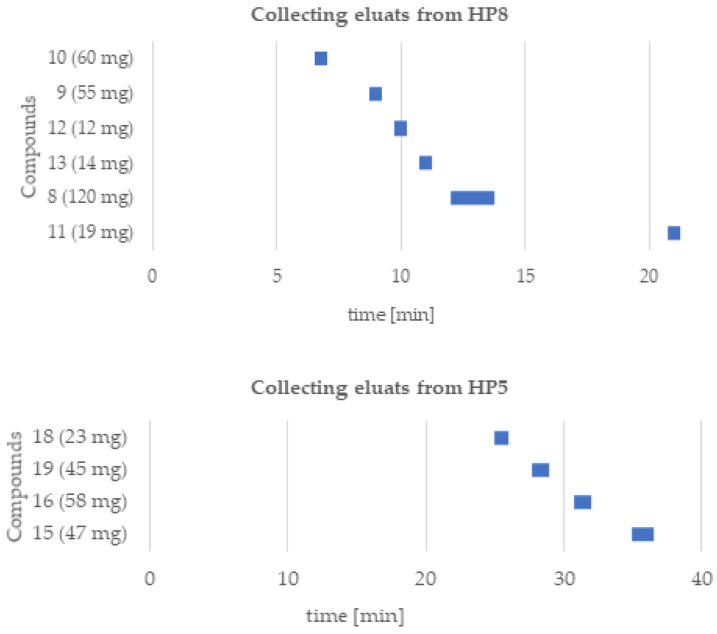
Time windows for collecting eluates from purification of chloroform fraction (**HP8**) and *n*-butanol fraction (**HP5**) by preparative HPLC.

The **HP8** extract (7 g) was dissolved in the necessary volume of methanol:DMSO solution (9:1) and then fractionated on a self-packed SPE column with C18-modified silica gel. Elution was performed with a water–methanol mixture in 4 steps (25%, 50%, 75%, and 100% methanol, 300 mL per step). Based on the HPLC analysis, fraction 3 (75%) was selected for isolation by the preparative HPLC technique. This process provided the following compounds: **10** (60 mg, t_R_ = 6.5–7 min), **9** (55 mg, t_R_ = 8.75–9.25 min), **12** (12 mg, t_R_ = 9.75–10.25 min), **13** (14 mg, t_R_ = 10.75–11.25 min), **8** (120 mg, t_R_ = 12–13.75 min), and **11** (19 mg, t_R_ = 20.75–21.25 min). In a subsequent step, the obtained eluate was reduced under vacuum and recrystallized (water:ethanol (5:95, *v*/*v*)). The **HP5** fraction was dissolved in a mixture of 10% methanol and then subjected to a self-packed C18 SPE column (25 g). Elution was carried out with a mixture of water (A) and methanol (B), with the B content increasing in steps (10–100% B, 200 mL per step). Based on the HPLC analysis, the combined fractions of 2–3 (20–30%) constituted a homogeneous component of **14** (108 mg) with >98% HPLC purity. After being eluted out in succession, fractions 4–5 (40–50%) were combined and selected for further purification using preparative HPLC. Pooled eluates were evaporated from residual organics and lyophilized to yield homogeneous compounds labeled compounds **18** (23 mg, t_R_ = 25–26 min), **19** (45 mg, t_R_ = 27.75–29 min), **16** (58 mg, t_R_ = 30.75–32 min), and **15** (47 mg, t_R_ = 35–36.5 min). The elution time windows of the compounds and their yields (mg) are shown in Figure 4.

#### 3.3.3. Hydrolysis of the Glycosides

Acid hydrolysis was performed as previously described by Mabry [13] with the following modifications. Samples of the glycosides were refluxed under acidic conditions for 2–6 h instead of 1 h. Subsequently, HCl (hydrochloric acid) residue was removed under a vacuum by adding portions of methanol. The aqueous residue was extracted three times with diethyl ether, dried under anhydrous Na_2_SO_4_ (sodium sulfate), evaporated, and analyzed as a methanolic solution along with the aglycone standards apigenin, kaempferol, and quercetin [23] by LC-PDA-MS. The aqueous layer was evaporated to dryness, and the residue was dissolved in methanol and analyzed with monosaccharide and uronic acid standards by cellulose TLC (thin-layer chromatography) (ethanol:ammonia:water, 20:1:4, *v*/*v*; derivative agent: aniline phthalate spray solution). The values of monosaccharide spots were observed as retention factors (Rfs) after aniline phthalate derivatization (110 °C, 10 min) as follows: L-arabinose, Rf = 0.48; D-xylose, Rf = 0.63; D-galactose, Rf = 0.34; D-glucose, Rf = 0.45; L-rhamnose, Rf = 0.77; glucuronic acid, Rf = 0.3; and galacturonic acid, Rf = 0.1.

### 3.4. Elucidation of Chemical Structure of Compounds ***8–19***

5,6,2’,6’-tetramethoxyflavone (zapotin) (**8**): grayish white, matte crystals, UV λ_max_ nm: 231, 258.7*sh* (shift), 327; +NaOMe: 237, 258.7*sh*, 327; +AlCl_3_: 230, 258*sh*, 328 nm; +NaOAc: 228, 265*sh*, 325; +H_3_BO_3_: 230, 263*sh*, 324. HPLC Rt min = 57.06. HRMS *m*/*z* = 344.1251 [M+H]+ (calculated for C_19_H_18_O_6_), difference = −1.01 ppm. Purity 98% by HPLC. The ^1^H-NMR and ^13^C-NMR results agree with the data provided in the literature [24].

5,2′-Dihydroxyflavone 2′-*O*-*β*-glucopyranoside (**9**): white, amorphous powder, (mp.: 259, 1–262 °C); [α]_D_: −34 (DMSO; c = 0.1); UV λ_max_ nm: 268, 326; +NaOMe: 274, 308*sh*, 386; +AlCl_3_: 284, 329, 489; +NaOAc: 267, 325; +H_3_BO_3_: 268, 325. HPLC Rt min = 48.87. HRMS *m*/*z* = 417.1187 [M+H]^+^ (calculated for C_21_H_20_O_9_), difference = 1.81 ppm. Purity 98% by HPLC. Predicted acidic pKa = 8.75; logP = −0.26 and logS = −3.84. The NMR data are provided in Table 1 and Figures S1–S7.

5,6-Dihydroxyflavone 6-*O*-(6″-*O*-*β*-glucopyranosyl)-β-glucopyranoside (hottonioside A) (**10**): yellow, amorphous powder, (mp.: 178–179,5 °C); [α]_D_: −66,18 (DMSO; c = 0.1); UV λ_max_ nm: 279, 310*sh* (shift), 345; +NaOMe: 280, 329*sh*, 391; +AlCl_3_: 301, 332*sh*, 408 nm; +NaOAc: 279, 308*sh*, 350; +H_3_BO_3_: 278, 308*sh*, 342. HPLC Rt min = 44.11. HRMS *m*/*z* = 577.1561 [M−H]^−^ (calculated for C_27_H_30_O_14_), difference = 0.11 ppm. Purity 98% by HPLC. Predicted acidic pKa = 9.09; logP = −2.38 and logS = −3.36. The NMR data are provided in Table 1 and Figures S8–S14.

5,2′ -Dihydroxyflavone (**11**): pale-yellow needle crystal, UV λ_max_ nm: 268, 340; +NaOMe: 248, 267*sh*, 398; +AlCl_3_: 278*sh*, 289, 349, 383; +NaOAc: 266, 346, 405; +H_3_BO_3_: 268, 340. HPLC Rt min = 66.27. HRMS *m*/*z* = 255.0506 [M−H]^−^ (calculated for C_15_H_10_O_4_), difference = 1.25 ppm. Purity 98% by HPLC. The ^1^H-NMR and ^13^C-NMR results agree with the data provided in the literature [25,26,27,28].

5,6-Dihydroxyflavone (**12**): yellow, amorphous powder, UV λ_max_ nm: 283, 359; +NaOMe: 298, 416; +AlCl_3_: 306, 335*sh*, 425; +NaOAc: 284, 380; +H_3_BO_3_: 284, 380. HPLC Rt min = 54.54. HRMS *m*/*z* = 253.0506 [M−H]^−^ (calculated for C_15_H_10_O_6_), difference = 2.21 ppm. Purity 98% by HPLC. For NMR spectral data, see [29].

5,7-Dihydroxyflavone (chrysin) (**13**): yellow, amorphous powder, UV λ_max_ nm: 247*sh* (shift), 268, 313; +NaOMe: 287, 262*sh*, 278, 361; +AlCl_3_: 250, 279, 331, 380; +NaOAc: 278, 360; +H_3_BO_3_: 269, 315. HPLC Rt min = 54.9. HRMS *m*/*z* = 253.0511 [M−H]^−^ (calculated for C_15_H_10_O_4_), difference = 2.03 ppm. Purity 98% by HPLC. For NMR spectral data, see [30].

Apigenin 7-*O*-(2″-*O*-*β*-glucuronide)-*β*-glucopyranoside (**14**): light-brown, amorphous powder, (mp.: 219–221 °C); [α]_D_: −56,53 (DMSO; c = 0.1); UV λ_max_ nm: 270, 332; +NaOMe: 271, 305, 343sh, 385; +AlCl_3_: 277, 300, 347, 385; +NaOAc: 269, 345, 394; +H_3_BO_3_: 270, 336. HPLC Rt min = 19.87. HRMS *m*/*z* = 607.13 [M−H]^−^ (calculated for C_27_H_28_O_16_), difference = −0.49 ppm. Purity 98% by HPLC. Predicted acidic pKa = 2.98; logP = −2.26 and logS = −3.04. The NMR data are provided in Table 1 and Figures S15–S21.

Kaempferol 3-*O*-(6″-*O*-*α*-rhamnopyranosyl)-*β*-glucopyranoside (nicotiflorin) (**15**): yellow powder UV λ_max_ nm: 267, 350; +NaOMe: 276, 327*sh*, 401; +AlCl_3_: 275, 305*sh*, 353, 398; +NaOAc: 275, 304*sh*, 369; +H_3_BO_3_: 267, 353. HPLC Rt min = 35.11. HRMS *m*/*z* = 593.1514 [M−H]^−^ (calculated for C_27_H_30_O_15_), difference = 0.39 ppm. Purity 98% by HPLC. The ^1^H-NMR and ^13^C-NMR results agree with the data provided in the literature [31].

Kaempferol-3-*O*-(6″-*O*-*β*-xylopyranosyl)-*β*-glucopyranoside (**16**): yellow, amorphous powder, UV λ_max_ nm: 268, 351; +NaOMe: 276, 328*sh*, 402; +AlCl_3_: 276, 306*sh*, 353*sh*, 400; +NaOAc: 275, 305*sh*, 376; +H_3_BO_3_: 268, 353. HPLC Rt min: 29.56. HRMS *m*/*z* = 579.1356 [M−H]^−^ (calculated for C_26_H_28_O_15_), difference = 0.29 ppm. Purity 98% by HPLC. Predicted acidic pKa = 7.62; logP = −2.99 and logS = −2.68. The NMR data are provided in Table 1 and Figures S22–S28.

Kaempferol (**17**): yellow, amorphous powder, UV λ_max_ nm: 253*sh*, 266, 322*sh*, 368; +NaOMe: 281, 321, 421; +AlCl_3_: 270, 305, 352, 425; +NaOAc: 273, 307,377; +H_3_BO_3_: 268, 324*sh*, 366. HPLC Rt min: 51.53. HRMS *m*/*z* = 285.0407 [M−H]^−^ (calculated for C_15_H_10_O_6_), difference = −0.12 ppm. Purity 98% by HPLC. The ^1^H-NMR and ^13^C-NMR results agree with the data provided in the literature [27].

Quercetin 3-*O*-(6″-*O*-*β*-xylopyranosyl)-*β*-glucopyranoside (**18**): yellow, amorphous powder, UV λ_max_ nm: 258, 300*sh*, 359; +NaOMe: 273, 332*sh*, 411; +AlCl_3_: 275, 307*sh*, 338*sh*, 434; +NaOAc: 272, 327*sh*, 375; +H_3_BO_3_: 262, 297*sh*, 379. HPLC Rt min: 22.69. HRMS *m*/*z* = 595.1316 [M−H]^−^ (calculated for C_26_H_28_O_16_), difference = 2.29 ppm. Purity 98% by HPLC. Predicted acidic pKa = 7.62; logP = −3.28 and logS = −2.18. Purity 98% by HPLC. The NMR data are provided in Table 1 and Figures S29–S35.

Quercetin 3-*O*-(6″-*O*-*α*-rhamnopyranosyl)-*β*-glucopyranoside (rutin) (**19**): yellow, amorphous powder, UV λ_max_ nm: 259, 300*sh*, 356; +NaOMe: 274, 331, 405; +AlCl_3_: 276, 305*sh*, 345*sh*, 427; +NaOAc: 271, 321, 369; +H_3_BO_3_: 264, 298, 375. HPLC Rt min: 25.57. HRMS *m*/*z* = 609.1460 [M−H]^−^ (calculated for C_27_H_30_O_16_), difference = 0.02 ppm. Purity 98% by HPLC. The ^1^H-NMR and ^13^C-NMR results agree with the data provided in the literature [31].

### 3.5. Conditions of Chromatographic Analysis

#### 3.5.1. Sample Preparation

A sample of the crude plant extract (20 mg/mL), as well as an isolated compound (1 mg/mL) and standards (1 mg/mL), were dissolved in DMSO, centrifuged, and then filtered through a 0.2 µm PVDF membrane. Then, samples were diluted in the initial mobile (95:5, ultra-pure water:acetonitrile) phase (1:10, *v*/*v*). All samples were prepared just before analysis.

#### 3.5.2. LC-PDA-HRMS Data Acquisition

The analysis was performed using a 1260 Infinity LC system consisting of a binary pump, a column oven, and a photo-diode array (PDA) detector combined with a 6230 LC/TOF mass spectrometer equipped with electrospray ionization (Agilent, Santa Clara, CA, USA). The autosampler was programmed to inject 5 µL of each sample. Separation of the analytes was carried out using a Kinetex XB-C18 (150 × 2.1 mm ID, 1.7 µm particle, batch No.: 5605-0180) column (Phenomenex, Torrance, CA, USA) guarded by a pre-column and maintained at 15 °C. The mobile phase ran at a flow rate of 0.2 mL/min with a run time of 110 min using the following gradient program (phase mobile A: ultra-pure water; phase mobile B: acetonitrile, both with 10 mM ammonia acetate buffer, pH 4.8): t = 0–3 min, 95:5; t = 10 min, 84:16; t = 38 min, 82:18; t = 45 min, 62:38; t = 72 min, 60:40; t = 100–103 min, 20:80 (mobile phase A:B), with a 7 min equilibration time. The PDA detector recorded spectra over a range of 190–600 nm, and the UV–Vis chromatogram was conducted at 270, 340, and 360 nm to match the values of the most intense absorption for polyphenols and methoxyflavones, flavones, and flavonols, respectively.

The ESI parameters were set as follows: a capillary voltage of 2.5 kV for negative mode with a nozzle voltage of 1 kV; a drying and sheath gas temperature of 350 °C; and a drying and sheath gas flow rate of 11 L/min. The nebulizer was set at 60 psi. The time-of-flight (TOF) MS was operated in high-resolution mode. The data were acquired over a mass range of *m*/*z* 120–1700. The Mass Hunter Qualitative Analysis 10.0 (Agilent, Santa Clara, CA, USA) software was used to complete and process the chromatographic data. Compounds were characterized based on the maxima observed in their UV–Vis spectra, as well as their MS spectra and retention times.

#### 3.5.3. Preparative HPLC

The analysis was conducted using an LC 1260 Infinity system equipped with a 1260 FC-AS G1364C fraction collector. The system was operated under the control of Open Lab CDS 2.7 software (Agilent, Santa Clara, CA, USA). The separation was optimized on a Luna C18(2) (150 × 4.6 mm ID, 5 µm particle, batch No.: 5291-0217) column (Phenomenex, Torrance, CA, USA) guarded by a pre-column. Isolation was performed using a Luna C18(2) (150 × 10 mm ID, 5 µm particle, batch No.: 5291-0227) column (Phenomenex, Torrance, CA, USA) that was fitted with a pre-column. Parameters were scaled up according to the increase in the column diameter. The collection of fractions was carried out on the basis of fixed time windows considering the latency and threshold recorded at 280 and 340 nm. For the optimized separation of **HP5**, a two-step gradient was used: 0–5 min at 10% B, followed by 45 min at 20% B extended for equilibration. A flow rate of 0.6 mL/min was used. For the **HP8** fraction, an isocratic of 45% B was used. In both cases, the mobile phase was ultra-pure water (A) and acetonitrile (B) with 0.1% formic acid. The thermostat temperature was set at 25 °C. Parameters were scaled up according to the increase in the column diameter.

### 3.6. Quantitative Characteristics of H. palustris Extracts

#### 3.6.1. Determination of the Total Phenol Content (TPC)

The total phenolic content (TPC) was determined using the Folin–Ciocalteu method [32]. In this method, 80 µL of extract or fraction solution (1 mg/mL), 80 µL of Folin–Ciocalteu (9:1, *v*/*v*) reagent, and 80 µL of 10% Na_2_CO_3_ (sodium carbonate) were mixed and incubated for 1 h at room temperature. The absorbance was measured at 630 nm (each measurement was repeated three times). The TPC is expressed in pyrogallol equivalents from the calibration curve.

#### 3.6.2. Determination of the Total Flavonoid Content (TFC)

The total flavonoid content (TFC) was determined using a previously described [33] method with Folin–Ciocalteu reagent, in which 50 µL of the sample was mixed with 10 µL of 10% AlCl_3_, 150 µL of 96% ethanol, and 10 µL of 1M CH_3_COOH. The absorbance was measured at 415 nm after 1 h of incubation at room temperature, and the values are expressed as luteolin equivalents. All tests were performed in triplicate.

#### 3.6.3. Determination of Total Phenolic Acid Content (TPAC)

The total phenolic acid content (TPAC) was determined spectrophotometrically using Arnov’s reagent [29]. In this method, 30 µL of the sample, 150 µL of distilled water, 30 µL of 0.5 M HCl, 30 µL of Arnov’s reagent, and 30 µL of 1 M NaOH were mixed into a clear, flat-bottomed 96-well plate. The absorbance of the resulting solution was measured at 490 nm. The TPAC was determined using a standard curve with caffeic acid.

#### 3.6.4. Determination of the Total Tannin Content (TTC)

The total tannin content (TTC) was examined using a previously described method [33] based on the difference between TPC values. Briefly, 1 mL of the sample was shaken with 10 mg of leather powder and then percolated. Afterwards, the filtrate (80 µL) was mixed with Folin–Ciocalteu (9:1, *v*/*v*) reagent (80 µL) and 10% sodium carbonate (80 µL). After 1 h of incubation, the absorbance was measured at 630 nm. Each assay was repeated in triplicate, and the results are presented as pyrogallol equivalents.

### 3.7. Antioxidant Activity (DPPH, ABTS, CUPRAC, FRAP)

#### 3.7.1. Free Radical Scavenging Activity (DPPH Assay)

The methanolic solutions of fractions or compounds (130 µL) were added to the DPPH solution (70 µL). The sample’s absorbance was recorded after 30 min of incubation in darkness. All tests were performed in triplicate [33]. The radical scavenging activity of the tested samples was calculated using an equation obtained through linear regression of the Trolox standard curve. The methanolic solutions of fractions or compounds (130 µL) were added to the DPPH solution (70 µL). The sample absorbance was recorded after 30 min of incubation in darkness. All tests were performed in triplicate [33]. The radical scavenging activity of the tested samples was calculated using the equation obtained through linear regression of the Trolox standard curve.

#### 3.7.2. ABTS [2,2′-azino-bis(3-ethylbenzothazoline-6-sulphonic acid)] Radical Cation Scavenging Activity

The potential inhibition of the production of the radical cation was measured using an Antioxidant Assay Kit. In short, the mixed solution, including 10 µL of the test sample, 20 µL of the myoglobin working solution, and 150 µL of the ABTS working solution, was incubated for 5 min. Then, 100 µL of the stop solution was added, and the absorbance was measured at 405 nm. All tests were performed in triplicate. The results were expressed as µM of Trolox equivalents obtained from the standard curve.

#### 3.7.3. Ferric-Reducing Antioxidant Power Method (FRAP)

The ferric-reducing power was measured using the Ferric-Reducing Antioxidant Power Assay Kit. In brief, 10 µL of the tested solution was added to 190 µL of the reaction solutions (containing FRAP Assay Buffer, FeCl_3_ solution, and FRAP Probe). All of the absorbance values were recorded at 594 nm after 1 h of incubation at 37 °C. The chelating activity was determined using a standard curve and evaluated as mM of ferrous ion equivalents. All tests were performed in triplicate.

#### 3.7.4. Cupric Ion Reducing Method (CUPRAC)

The total antioxidant capacity was determined using an Antioxidant Assay Kit MAK334. In short, 20 µL of each fraction or compound solution was added to 100 µL of the Reaction Mix in the clear, flat-bottomed 96-well plate. The absorbance was measured at 570 nm after 10 min of incubation at room temperature. The results were determined using a standard curve evaluated for Trolox.

### 3.8. Acetylcholinesterase Inhibitory Activity

Acetylcholinesterase inhibition was determined according to the previously described method [34] with a slight modification. The assay was carried out in Tris-HCl buffer (pH = 8 at 25 °C). The test extracts or compounds were diluted in the same buffer to the appropriate concentrations. Firstly, the reaction mixture containing the sample solution (50 µL), AChE solution (25 µL, 0.25 U/mL), and Ellman’s Reagent (3 mM, 125 µL) was incubated for 15 min at room temperature. Subsequently, 25 µL of ACh iodide (15 mM) was added, and after a further quarter-hour of incubation, the absorbance was estimated at 405 nm. The blank well contained the same amount of Tris-HCl buffer instead of the sample solution. Galantamine was used as a positive control. All measurements were conducted in triplicate, and the values are presented as IC_50_ means with standard deviations (SDs). The percent inhibition was calculated using the following formula:

Enzyme inhibition activity (%) = (B − S)/B × 100%

where B is the blank, and S is the sample absorbance.

## 4. Statistical Analysis and Simulated Data

All results from the enzyme inhibitory tests are presented as the mean ± standard deviation (SD) from at least three independent replicates. Calculations for the biological analysis were performed using GraphPad Prism 9 software (Trial, GraphPad Software, San Diego, CA, USA) and MS Excel 2019 (Microsoft, Redmond, WA, USA). Statistical differences and linear regression parameters for the standard curve were established using ANOVA with verification of the statistical significance. The physico-chemical properties, pKa, log P, and log S values, were calculated using Chemaxon’s Protonation software (Basel, Switzerland).

## 5. Conclusions

In our study, twelve flavonoid compounds were isolated from the aerial parts of *H. palustris*, three of which are new natural products and two were comprehensively characterized for the first time. In addition, the qualitative analysis showed the presence of 31 secondary metabolites of *H. palustris*, which included phytoconstituents characteristic of the Primulaceae family, such as saponins and flavone derivatives, polymethoxyflavones. Quantitative analysis showed a high content of polyphenols in the analyzed fractions and extracts. All tested samples were characterized by high antioxidant potential. The ethyl acetate fraction characterized by a strong ability to inhibit acetylcholinesterase was also described, while the tested compounds showed a moderate inhibitory effect. The most active compound turned out to be dibenzoylmethane. In summary, the chemical composition of the *H. palustris* herb resembles the well-known representatives of the Primulaceae family, as evidenced by the presence of the described chemophenetic markers. In addition, after additional toxicological analysis, it may be a good source of very rare polyphenolic compounds with a protective effect against oxidative stress and its degenerative effects on sensitive tissues.

## Figures and Tables

**Table 1:** The ^13^C and ^1^H spectral data of compounds **9, 10, 14, 16,** and **18** (400 MHz for ^1^H and 100 MHz for the ^13^C spectrum; δ in *ppm*, *J* in *Hz*, DMSO-*d*6).

C No.	9		10		14		16		18	
	*δ* C	*δ* H	*δ* C	*δ* H	*δ* C	*δ* H	*δ* C	*δ* H	*δ* C	*δ* H
**2**	161.85 ^C^	-	164.58 ^C^	-	164.26 ^C^	-	157 ^C^	-	156.19 ^C^	-
**3**	110.78 ^B^	7.16, s	104.97 ^B^	7.03, s	103.05 ^B^	6.85, s	133.69 ^C^	-	133.26 ^C^	-
**4**	183.29 ^C^	-	183.85 ^C^	-	182.06 ^C^	-	177.86 ^C^	-	177.23 ^C^	-
**5**	159.8 ^C^	-	148.51 ^C^	-	161.13 ^C^	-	161.64 ^C^	-	161.14 ^C^	-
**6**	110.69 ^B^	6.82, d, *J* = 8.16	140.59 ^C^	-	99.54 ^B^	6.45, s	99.19 ^B^	6.19, s	98.88 ^B^	6.15, s
**7**	135.9 ^B^	7.68, t, *J* = 8.28, 8.16	107.11 ^B^	7.72, d, *J* = 9.03	162.76 ^C^	-	164.69 ^C^	-	164.94 ^C^	-
**8**	107.53 ^B^	7.38, d, *J* = 8.28	124.14 ^B^	7.18, d, *J* = 9.03	95.25 ^B^	6.82, s	94.21 ^B^	6.41, s	93.7 ^B^	6.35, s
**9**	156.2 ^C^	-	150.49 ^C^	-	156.83 ^C^	-	156.93 ^C^	-	156.41 ^C^	-
**10**	110.03 ^C^	-	110.61 ^C^	-	105.4 ^C^	-	104.49 ^C^	-	103.72 ^C^	-
**5-OH**		12.71, s	-	12.73, s	-	12.96, s	-	12.59, s	-	12.61, s
**4′-OH**		-	-	-	-	10.44, s	-	10.24, s	-	nd
**1′**	120.07 ^C^	-	130.84 ^C^	-	121.04 ^C^	-	121.35 ^C^	-	121.08 ^C^	-
**2′**	155.63	-	126.84 ^B^	8.09, d, *J* = 7.03	128.61 ^B^	7.95, d, *J* = 8.53	131.42 ^B^	8.03, d, *J* = 8.66	116.15 ^B^	7.56, s
**3′**	115.59 ^B^	7.17, d, *J* = 6.78 ^A^	129.46 ^B^	7.6, m	115.99 ^B^	6.94, d, *J* = 8.53	115.57 ^B^	6.88, d, *J* = 8.66	144.82 ^C^	-
**4′**	133.23 ^B^	7.58, t, *J* = 7.4, 7.4	132.58 ^B^	7.6, m	161.36 ^C^	-	160.4 ^C^	-	148.6 ^C^	-
**5′**	122.04 ^B^	7.23, t, *J* = 7.4, 7.4	129.46 ^B^	7.6, m	115.99 ^B^	6.94, d, *J* = 8.53	115.57 ^B^	6.88, d, *J* = 8.66	115.24 ^B^	6.83, d, *J* = 8.03
**6′**	129.26 ^B^	7.94, d, *J* = 6.78	126.84 ^B^	8.09, d, *J* = 7.03	128.61 ^B^	7.95, d, *J* = 8.53	131.42 ^B^	8.03, d, *J* = 8.66	121.66 ^B^	7.58, d, *J* = 2.26
	***2′-O-glucosyl***		***6-O-glucosyl***		***7-O-glucosyl***		***3-O-glucosyl***		***3-O-glucosyl***	
**1″**	100.27	5.13, d, *J* = 7.03 ^B^	101	4.92, d, *J* = 6.53 ^B^	98.29	5.2, d, *J* = 7.03 ^B^	101.48	5.37, d, *J* = 7.53 ^B^	100.94	5.37, d, *J* = 7.28 ^B^
**2″**	73.33	3.31 ^A,B^	73.81	2.96 ^A,B^	82.74	3.54 ^A,B^	73.61	2.85 ^A,B^	73.98	3.21 ^A,B^
**3″**	77.21	3.46 ^A,B^	76.96	3.05 ^A,B^	75.76	3.15 ^A,B^	76.73	3.22 ^A,B^	76.35 ^A^	3.24 ^A,B^
**4″**	69.56	3.2 ^A,B^	70.31	3.12 ^A,B^	69.14	3.26 ^A,B^	70.13	3.13 ^A,B^	69.76	2.98 ^A,B^
**5″**	76.72	3.32 ^A,B^	76.80	3.33 ^A,B^	76.98	3.5 ^A,B^	76.64	2.85 ^A,B^	76.21	2.87 ^A,B^
**6″**	60.59	3.72, d, *J* = 11.54	68.76	3.99, d, *J* = 9.79	60.48	3.73, d, *J* = 9.79	68.35	3.79, d, *J* = 11.29	69.41	3.81, d, *J* = 10.79
		3.49, d, *J* = 5.27 ^B^		3.62 ^A,B^		3.57 ^A,B^		3.45 ^A,B^		3.44 ^A,B^
	-		***6″-O-glucosyl***		***glucuronide moiety***		***2″-O-xylosyl***		***2″-O-xylosyl***	
**1″′**	-	-	103.5 ^B^	4.2, d, *J* = 7.78	104.44 ^B^	4.55, d, *J* = 7.78	104.13 ^B^	4.0, d, *J* = 7.28	103.65 ^B^	4.01, d, *J* = 7.28
**2″′**	-	-	73.43	3.29 ^A,B^	74.25	3.04 ^A,B^	74.55	3.16 ^A,B^	73.13	2.82 ^A,B^
**3″′**	-	-	76.82	3 ^A,B^	75.55	3.71 ^A,B^	76.7 ^A,D^	3.22 ^A,B^	76.35 ^A^	3.24 ^A,B^
**4″′**	-	-	70.02	3.30 ^A,B^	71.69	3.24 ^A,B^	69.86	2.81 ^A,B^	69.41	3.27 ^A,B^
**5″′**	-	-	76.16	3.61 ^A,B^	75.62	3.49 ^A,B^	65.79	3.51 ^A,B^	65.32	3.54 ^A,B^
								2.76 ^A,B^		2.75 ^A,B^
**6″′**	-	-	61.24	3.67 ^A,B^	170.67	-	-	-	-	-
				3.41 ^A,B^						

^A^—signals or overlapping multiplets; ^B^—chemical shift based on the HSQC/HMQC spectrum; ^C^—chemical shift based on the HMBC spectrum; ^D^—chemical shift based on the DEPT spectrum (see Figure S36).

**Table 2:** The UV–Vis and MS data of compounds identified in **HP1–HP8** by liquid chromatography–photodiode detection array–high-resolution mass spectrometry (LC-PDA-HRMS).

No	Rt [min]	λ Max [nm]	Observed ^#^	Formula	Δ [ppm]	Fragmentation	Compounds	Presence in HP							
						Negative/Positive		HP1	HP2	HP3	HP4	HP5	HP6	HP7	HP8
P1	17.35	256, 266, 348	623.1267 ^A^	C_27_H_28_O_17_	2.26	**623**, 447, 285/**625**, 287	kaempferol hex-uro derivative	x	x	x		x			
P2	19.87	268, 336	607.13 ^A^	C_27_H_28_O_16_	−0.49	**607**, 269/**609**, 271	apigenin 7-*O*-(2″-*O*-*β*-gluc)-*β*-glu (**14**)	x	x	x	x	x			x
P3	22.27	254, 266, 295, 354	595.1317 ^A^	C_26_H_28_O_16_	2.29	**595**, 300/**597**, 465, 303	quercetin pent-hex	x	x			x			x
P4	22.69	254, 266, 295, 354	595.1316 ^A^	C_26_H_28_O_16_	2.29	**595**, 300/**597**, 465, 303	quercetin 3-*O*-(6″-*O*-*β*-xyl)-*β*-glu (**18**)	x	x		x	x			x
P5	26.10	256, 266, 295, 354	609.146 ^A^	C_27_H_30_O_16_	0.02	**609**, 300/**611**, 465, 303	rutin (**19**)	x	x	x	x	x			x
P6	28.83	256, 264, 292, 352	579.1355 ^A^	C_26_H_28_O_15_	3.6	**579**, 284/**581**, 417, 287	kaempferol pent-hex	x	x		x	x			x
P7	29.56	256, 264, 292, 352	579.1356 ^A^	C_26_H_28_O_15_	0.29	**579**, 284/**581**, 417, 287	kaempferol 3-*O*-(6″-*O*-*β*-xyl)-*β*-glu (**16**)	x	x		x	x			x
P8	35.11	266, 294, 348	593.1514 ^A^	C_27_H_30_O_15_	0.39	**593**, 284/**595**, 449, 287	nicotiflorin (**15**)	x	x	x	x	x			x
P9	44.11	278, 314*sh*, 352*sh*	577.1561 ^A^	C_27_H_30_O_14_	0.11	**577**, 253/**579**, 417, 255	hottonioside A (**10**)				x	x			x
P10	46.63	278, 314*sh*, 352*sh*	577.1565 ^A^	C_27_H_30_O_14_	−0.76	**577**, 254/**579**, 255	dihydroxyflavone hex-hex				x				x
P11	48.65	257, 368	301.0354 ^A^	C_15_H_10_O_7_	3.31	301/303	quercetin^S^	x	x	x					
P12	48.87	268, 324	417.1187 ^B^	C_21_H_20_O_9_	1.81	-/**417**, 255	2′,5-dihydroxyflavone 2′-*O*-*β*-glu (**9**)				x			x	x
P13	50.16	268, 290*sh*, 336	269.0457 ^A^	C_15_H_10_O_5_	0.69	269/271	apigenin^S^	x	x	x	x			x	x
P14	51.53	250, 265, 295*sh*, 320*sh*, 365	285.0407 ^A^	C_15_H_10_O_6_	0.12	285/287	kaempferol (**17**)	x	x	x	x			x	x
P15	53.46	nd	1249.6214 ^A^	C_60_H_98_O_27_	−0.19	**1249**, 328/-	sakurasosaponin		x	x		x			x
P16	54.12	nd	1103.5664 ^A^	C_54_H_88_O_23_	1.53	**1103**, 328/-	primulasaponin 1		x	x		x			x
P17	54.36	nd	1219.612 ^A^	C_54_H_88_O_23_	1.53	1219/-	triterpene saponin derivative		x	x		x			x
P18	54.54	282, 318, 360	253.0506 ^A^	C_15_H_10_O_4_	2.21	253/255	5,6-dihydroxyflavone (**12**)						x	x	x
P19	54.69	nd	957.5111 ^A^	C_41_H_82_O_24_	−1.1	957/-	triterpene saponin derivative		x			x			
P20	54.9	272, 302, 334	253.0511 ^A^	C_15_H_10_O_4_	2.03	253/255	chrysin (**13**)						x	x	x
P21	57.06	230, 258, 335	344.1251 ^B^	C_19_H_18_O_6_	−1.01	-/**343**, 328, 313, 299	zapotin (**8**)	x	x	x			x	x	x
P22	59.29	258, 333	313.0718 ^A^	C_17_H_14_O_6_	0.67	**313**, 298, 283/315	5,6′-dihydroxy-2′,3′-dimethoxyflavone (**7**)	x	x	x			x	x	x
P23	59.95	259, 327	283.0612 ^A^	C_16_ H_12_ O_5_	0.82	**283**, 268/285	2’,5-dihydroxy-6-methoxyflavone (**6**)	x	x	x			x	x	x
P24	66.27	268, 340	255.0506 ^A^	C_15_H_10_O_4_	1.25	253/255	2’,5-dihydroxyflavone (**11**)	x	x				x	x	x
P25	72.64	257, 331	327.0887 ^A^	C_18_H_16_O_6_	1.36	327/**329**, 299	5-hydroxy-2’,3’,6’-trimethoxyflavone (**5**)	x					x	x	x
P26	81.71	260, 327	297.0772 ^A^	C_17_H_14_O_5_	1.35	297/299	5-hydroxy-2’,6’-dimethoxyflavone (**4**)	x	x				x	x	x
P27	81.71	271, 336	239.0740 ^B^	C_15_H_10_O_3_	4.78	237/239	5-hydroxyflavone (**2**)	x	x				x	x	x
P28	84.87	260, 327	267.0663 ^A^	C_16_H_12_O_4_	0.93	267/**269**, 254	5-hydroxy-2′-methoxyflavone (**3**)	x	x				x	x	x
P29	91.94	252, 345	223.0765 ^A^	C_15_H_12_O_2_	3.2	223/225	1,3-diphenylpropane-1,3-dione (**1**)	x	x				x	x	x
P30	97.48	310, 340, 460	687.2346 ^A^	C_52_H_30_O_2_	1.02	**687**, 365/-	unknown		x						
P31	99.46	280, 325, 395, 410	591.2645 ^A^	C_34_H_40_O_9_	−3.65	591/631, **593**	unknown		x	x		x	x		x

#—exact mass registered in [M−H]^−^ (A) or [M+H]^+^ (B) ion; *sh*—peak shoulder; nd—not detected; Δ—error mass; bold—most abundant; S—reference substance; hex—hexose; pent—pentose; uro—uronide; glu—glucose; xyl—xylose; gluc—glucuronide acid.

**Table 3:** Phytochemical analysis of the total phenolic (TPC), total flavonoid (TFC), phenolic acid (TPAC), and tannin contents (TTC) of **HP1**–**HP8**.

Extracts	TPC	TFC	TPAC	TTC
	(mg P_eq_/g extract) ^A^	(mg L_eq_/g extract) ^B^	(mg CA_eq_/g extract) ^C^	(mg P_eq_/g extract) ^A^
**HP1**	64.58 ± 1.01	7.10 ± 0.41	1.02 ± 0.06	2.94 ± 0.12
**HP2**	49.54 ± 1.75	2.58 ± 0.16	0.58 ± 0.01	2.00 ± 0.31
**HP3**	12.44 ± 0.20	1.62 ± 0.07	0.51 ± 0.01	2.11 ± 0.46
**HP4**	67.81 ± 2.02	3.23 ± 0.29	0.80 ± 0.04	4.07 ± 0.82
**HP5**	51.60 ± 2.37	2.69 ± 0.05	0.64 ± 0.03	5.40 ± 0.86
**HP6**	64.52 ± 1.87	9.23 ± 0.12	3.03 ± 0.14	3.24 ± 0.58
**HP7**	62.12 ± 1.67	7.63 ± 0.36	3.48 ± 0.21	1.51 ± 0.28
**HP8**	58.31 ± 1.92	3.88 ± 0.12	0.76 ± 0.01	3.33 ± 0.82

^A^—expressed as pyrogallol equivalents (P_eq_); ^B^—expressed as luteolin equivalents (L_eq_); ^C^—expressed as caffeic acid equivalents (CA_eq_). All data are represented as the mean with standard deviation from triplicate measurements.

**Table 4:** Antioxidant and acetylcholinesterase inhibitory activities of **HP1–HP8**.

Extracts	DPPH ^A^	ABTS ^A^	FRAP ^B^	CUPRAC ^A^	AChE ^C^
	(µM Teq)	(µM Teq)	(mM Fe^2+^/mL)	(µM Teq)	IC_50_ (µg/mL)
**HP1**	409.98 ± 4.83	945.81 ± 4.03	2.63 ± 0.04	354.42 ± 2.01	274.15 ± 0.98
**HP2**	299.23 ± 3.59	774.72 ± 2.50	2.50 ± 0.04	375.08 ± 1.32	378.25 ± 3.22
**HP3**	384.66 ± 3.65	773.90 ± 3.69	1.55 ± 0.15	79.67 ± 0.32	na
**HP4**	452.16 ± 4.37	1132.21 ± 2.50	3.68 ± 0.21	385.19 ± 1.52	219.77 ± 0.37
**HP5**	298.18 ± 3.59	1001.30 ± 2.28	2.36 ± 0.17	399.26 ± 2.63	na
**HP6**	384.66 ± 1.83	1075.37 ± 3.71	4.35 ± 0.45	219.03 ± 0.76	260.24 ± 1.60
**HP7**	494.35 ± 4.83	1099.42 ± 3.25	4.69 ± 0.14	306.95 ± 2.75	282.46 ± 0.47
**HP8**	376.23 ± 3.48	945.81 ± 3.03	2.16 ± 0.06	129.79 ± 1.32	290.26 ± 1.51

^A^—expressed as Trolox equivalents (T_eq_) and ^B^—expressed as Fe^2+^/mL where all data are represented as the mean with standard deviation from triplicate measurements; ^C^—data are represented as the mean of the IC_50_ value with standard deviation from at least triplicate measurements; galantamine (IC_50_ = 91.23 ± 0.52 µg/mL) was used as a positive control; na—no significant activity.

**Table 5:** Antioxidant and acetylcholinesterase inhibitory activities of compounds **1–19**.

Compounds	DPPH ^A^	ABTS ^A^	FRAP ^B^	CUPRAC ^A^	AChE ^C^
	(µM Teq)	(µM Teq)	(mM Fe^2+^/mL)	(µM Teq)	IC_50_ (µM)
**1**	39.81 ± 2.76	300.12 ± 4.97	na	3.18 ± 0.32	144.83 ± 1.30
**2**	41.71 ± 2.28	317.47 ± 3.91	na	na	314.38 ± 1.72
**3**	39.39 ± 1.59	330.59 ± 4.04	na	na	279.70 ± 0.45
**4**	28.63 ± 2.40	326.76 ± 6.64	na	na	325.42 ± 3.21
**5**	34.32 ± 0.97	280.02 ± 1.55	na	na	284.61 ± 0.80
**6**	227.55 ± 2.85	781.28 ± 4.38	15.81 ± 0.85	366.29 ± 1.24	391.78 ± 1.06
**7**	37.85 ± 1.90	277.56 ± 2.88	na	na	390.83 ± 0.66
**8**	167.89 ± 1.08	756.95 ± 5.10	0.05 ± 0.01	na	464.45 ± 1.66
**9**	170.56 ± 2.49	204.59 ± 2.26	na	20.33 ± 1.57	399.72 ± 0.64
**10**	204.31 ± 3.31	624.94 ± 5.70	na	19.79 ± 1.03	376.11 ± 1.87
**11**	175.83 ± 2.83	661.30 ± 3.81	0.07 ± 0.01	44.50 ± 1.35	371.51 ± 0.66
**12**	1522.38 ± 7.31	1052.41 ± 5.51	54.25 ± 1.03	1060.42 ± 5.33	362.19 ± 0.39
**13**	227.51 ± 1.83	728.26 ± 1.08	0.08 ± 0.01	na	330.15 ± 1.77
**14**	341.42 ± 3.16	746.84 ± 1.85	1.63 ± 0.03	66.48 ± 0.56	417.78 ± 1.11
**15**	250.72 ± 1.65	702.29 ± 2.47	0.40 ± 0.06	56.81 ± 1.52	407.44 ± 1.41
**16**	300.29 ± 1.87	717.32 ± 3.47	0.67 ± 0.06	74.40 ± 1.49	364.34 ± 1.79
**17**	792.53 ± 7.31	1095.04 ± 1.85	36.87 ± 1.20	848.64 ± 3.49	276.90 ± 1.41
**18**	1336.75 ± 6.33	1147.52 ± 7.06	63.98 ± 1.75	1401.11 ± 2.64	302.85 ± 3.33
**19**	1467.53 ± 5.48	1149.16 ± 5.25	52.98 ± 1.35	1258.68 ± 3.49	445.32 ± 0.35

^A^—expressed as Trolox equivalents (T_eq_) and ^B^—expressed as Fe^2+/^mL, where all data are represented as the mean with standard deviation from triplicate measurements; ^C^—data are represented as the mean of IC_50_ value with standard deviation from at least triplicate measurements; galantamine (IC_50_ = 91.23 ± 0.52 µg/mL) was used as a positive control; na—no significant activity.

## Data Availability

Data are contained within the article and in the Supplementary Material.

## Supplementary Materials

The following supporting information can be downloaded at: https://www.mdpi.com/article/10.3390/molecules27228034/s1, Figure S1: ^13^C NMR spectrum (100 MHz) of compound **9** in DMSO-*d*6. Figure S2: Mass spectrum in positive ion mode (fragmentor = 180 V) of compound **9**. Figure S3: ^1^H NMR spectrum (400 MHz) of compound **9** in DMSO-*d*6. Figure S4: The UV spectrum of compound **9**. Figure S5: The COSY spectrum of compound **9** in DMSO-*d*6. Figure S6: The HSQC spectrum of compound **9** in DMSO-*d*6. Figure S7: The HMBC spectrum of compound **9** in DMSO-*d*6. Figure S8: Mass spectrum in negative ion mode (fragmentor = 180 V) of compound **10**. Figure S9: ^13^C NMR spectrum (100 MHz) of compound **10** in DMSO-*d*6. Figure S10: The UV spectrum of compound **10**. Figure S11: ^1^H NMR spectrum (400 MHz) of compound **10** in DMSO-*d*6. Figure S12: The COSY spectrum of compound **10** in DMSO-*d*6. Figure S13: The HMQC spectrum of compound **10** in DMSO-*d*6. Figure S14: The HMBC spectrum of compound **10** in DMSO-*d*6. Figure S15: ^13^C NMR spectrum (100 MHz) of compound **14** in DMSO-*d*6. Figure S16: Mass spectrum in negative ion mode (fragmentor = 180 V) of compound **14**. Figure S17: The UV spectrum of compound **14**. Figure S18: ^1^H NMR spectrum (400 MHz) of compound **14** in DMSO-*d*6. Figure S19: The COSY spectrum of compound **14** in DMSO-*d*6. Figure S20: The HSQC spectrum of compound **14** in DMSO-*d*6. Figure S21: The HMBC spectrum of compound **14** in DMSO-*d*6. Figure S22: Mass spectrum in negative ion mode (fragmentor = 180 V) of compound **16**. Figure S23: ^13^C NMR spectrum (100 MHz) of compound 16 in DMSO-*d*6. Figure S24: The UV spectrum of compound **16**. Figure S25: ^1^H NMR spectrum (400 MHz) of compound **16** in DMSO-*d*6. Figure S26: The COSY spectrum of compound **16** in DMSO-*d*6. Figure S27: The HMQC spectrum of compound **16** in DMSO-*d*6. Figure S28: The HMBC spectrum of compound **16** in DMSO-d6. Figure S29: The UV spectrum of compound **18**. Figure S30: ^13^C NMR spectrum (100 MHz) of compound **18** in DMSO-*d*6. Figure S31: Mass spectrum in negative ion mode (fragmentor = 180 V) of compound **18**. Figure S32: ^1^H NMR spectrum (400 MHz) of compound **18** in DMSO-*d*6. Figure S33: The COSY spectrum of compound **18** in DMSO-*d*6. Figure S34: The HMQC spectrum of compound **18** in DMSO-*d*6. Figure S35: The HMBC spectrum of compound **18** in DMSO-*d*6. Figure S36: The DEPT spectrum of compound **16** in DMSO-*d*6. Figure S37: The UV chromatogram of extract **HP1** obtained by LC-PDA-HRMS. Figure S38: The UV with crucial MS chromatogram of extract **HP2** obtained by LC-PDA-HRMS. Figure S39: The UV with crucial MS chromatogram of extract **HP3** obtained by LC-PDA-HRMS. Figure S40: The UV chromatogram of fraction **HP4** obtained by LC-PDA-HRMS. Figure S41: The UV with crucial MS chromatograms of fraction **HP5** obtained by LC-PDA-HRMS. Figure S42: The UV chromatogram of extract **HP6** obtained by LC-PDA-HRMS. Figure S43: The UV chromatogram of extract **HP7** obtained by LC-PDA-HRMS. Figure S44: The UV with crucial MS chromatograms of fraction **HP8** obtained by LC-PDA-HRMS.

## Sample Availability

Samples of compounds **8–19** are available from the authors.

## Abbreviations

UV	ultraviolet spectroscopy
HRMS	high-resolution mass spectrometry
NMR	nuclear magnetic resonance
2D NMR	two-dimensional nuclear magnetic resonance
COSY	correlation spectroscopy
HSQC	heteronuclear single quantum coherence
HMBC	heteronuclear multiple bond correlation
DEPT	distortionless enhancement by polarization transfer
UV–Vis	ultraviolet–visible spectroscopy
TPC	total phenolic content
TFC	total flavonoid content
TPAC	total phenolic acid content
TTC	total tannin content
ABTS	2,2′-azino-bis(3-ethylbenzthiazoline-6-sulfonic acid)
FRAP	ferrous reducing antioxidant potential
DPPH	2,2-diphenyl-1-picrylhydrazyl
CUPRAC	cupric reducing antioxidant capacity
FeCl_3_	ferric (III) chloride
AChE	acetylcholinesterase
ACh iodide	acetylcholine iodide
SD	standard deviation
IC_50_	concentration at which a substance exerts half of its maximal inhibitory effect
