# Supplementary material for: In Vitro Screening for Anti-Acetylcholinesterase and Antioxidant Activities of Hottonia palustris L. Extracts and Their Unusual Flavonoids
Source: Molecules. 2022 Nov 19;27(22):8034. doi: 10.3390/molecules27228034 (PMC9692424; doi:10.3390/molecules27228034)
Supplement: Supplementary file 1 [file molecules-27-08034-s001.zip › molecules-2021776-supplementary.pdf]

molecules

Supplementary Materials

In Vitro Screening for Anti-Acetylcholinesterase and Antioxidant Activities of *Hottonia palustris* L. Extracts and Their Unusual Flavonoids

Jakub W. Strawa [1], Katarzyna Jakimiuk [1], Zuzanna Kita [2] and Michał Tomczyk [1,*]

[1] Department of Pharmacognosy, Faculty of Pharmacy with the Division of Laboratory Medicine, Medical University of Białystok, ul. Mickiewicza 2a, 15-230 Białystok, Poland
[2] Students' Scientific Association, Department of Pharmacognosy, Faculty of Pharmacy, Medical University of Bialystok, ul. Mickiewicza 2a, 15-230 Białystok, Poland
* Correspondence: michal.tomczyk@umb.edu.pl; Tel.: +48-85-748-56-94

Keywords: *Hottonia palustris*; Primulaceae; flavonoids; LC-HRMS; antioxidant activity; acetylcholinesterase inhibition

1. Chemicals and General Experimental Procedures

Diethyl ether, ethyl acetate, butan-1-ol, dimethyl sulfoxide, hydrochloric acid, formic acid and acetic acid, ethanol as well as modified silica gel Bakerbond C18 40 µm Prep LC were purchased from Avantor (Gliwice, Poland). Ammonia acetate was purchased from Merck (Darmstadt, Germany). Methanol was purchased from P.P.H. Stanlab (Lublin, Poland). Ammonium hydroxide was purchased Honeywell (Wabash, IN, USA). Sodium sulfate anhydrous was purchased from Chempur (Piekary Śląskie, Poland). Sephadex LH20 was purchased from GE Healthcare (Uppsala, Sweden). The adsorbent used for LPLC, silica gel 60 (0.063–0.2 mm), a TLC aluminum plate coated with silica gel 60 F254, and a TLC cellulose plate on glass support were purchased from Merck (Darmstadt, Germany). Acetonitrile Optima (LC/MS grade) was purchased from Fisher Scientific (Loughborough, UK). Ultrapure water was obtained using the POLWATER DL3-100 system (Kraków, Poland). Zapotin, 5, 2'-dihydroxyflavone were purchased from Biosynth-Carbosynth (Compton, UK). 5,6-Dihydroxyflavone was purchased from Thermo Scientific (Waltham, MA, USA). 5,7-Dihydroxyflavone was purchased from Cayman Chemical (Ann Arbor, MI, USA). Standards of the monosaccharides purchased from Merck (Darmstadt, Germany). D-glucuronic acid and D-galacturonic acid were purchased from Cayman Chemical (Ann Arbor, MI, USA). Aniline phthalate spray solution for TLC and polyamide for LPLC were purchased from Carl Roth (Karlsruhe, Germany). Extract preparation was carried out using an ultrasonic bath (40 kHz, Emmi-MF30, EMAG, Mörfelden-Walldorf, Germany). Solvent residues were removed by distillation (Rotavapor R-215 coupled with vacuum controller V-855 (Büchi, Flawil, Switzerland) and freeze-drying (Lymph-lock, Labconco, Kansas City, MO, USA). UV spectra were recorded by an Analytic Jena SPECORD 200 Plus instrument (Jena, Germany). Melting points were observed using a BÜCHI B-535 (Büchi, Flawil, Switzerland). NMR spectra were acquired using a Thermo Fisher Scientific Bruker Avance II 400 spectrometer (Billerica, MA, USA). Specific rotation [α] was recorded using a P-2000 digital polarimeter (Jasco, Hachioji, Tokyo, Japan) at 25 ± 0.5°C and at the sodium D line. Galantamine hydrobromide from *Lycoris* sp., TRIS hydrochloride, and phosphate buffer, AChE (acetylcholinesterase), Ellman's Reagent (5,5-dithio-bis-(2-nitrobenzoic acid)), ACh iodide (acetylcholine iodide), kits for antioxidant analyses were purchased from Sigma-Aldrich. Control and pH adjustments were made using a pH meter Hanna

edge (Woonsocket, RI, USA). Measurement of absorbance during the evaluation of the antioxidant activity and the inhibition of AchE activity was performed using a microplate reader EPOCH 2 (BioTek, Winooski, VT, USA).

2. **Obtained data**

Figure S1: ^{13}C NMR spectrum (100 MHz) of compound **9** in DMSO-*d*6.
Figure S2: Mass spectrum in positive ion mode (fragmentor = 180 V) of compound **9.**
Figure S3: ^{1}H NMR spectrum (400 MHz) of compound **9** in DMSO-*d*6.
Figure S4: The UV spectrum of compound **9.**
Figure S5: The COSY spectrum of compound **9** in DMSO-*d*6.
Figure S6: The HSQC spectrum of compound **9** in DMSO-*d*6.
Figure S7: The HMBC spectrum of compound **9** in DMSO-*d*6.
Figure S8: Mass spectrum in negative ion mode (fragmentor = 180 V) of compound **10.**
Figure S9: ^{13}C NMR spectrum (100 MHz) of compound **10** in DMSO-*d*6.
Figure S10: The UV spectrum of compound **10.**
Figure S11: ^{1}H NMR spectrum (400 MHz) of compound **10** in DMSO-*d*6.
Figure S12: The COSY spectrum of compound **10** in DMSO-*d*6.
Figure S13: The HMQC spectrum of compound **10** in DMSO-*d*6.
Figure S14: The HMBC spectrum of compound **10** in DMSO-*d*6.
Figure S15: ^{13}C NMR spectrum (100 MHz) of compound **14** in DMSO-*d*6.
Figure S16: Mass spectrum in negative ion mode (fragmentor = 180 V) of compound **14.**
Figure S17: The UV spectrum of compound **14.**
Figure S18: ^{1}H NMR spectrum (400 MHz) of compound **14** in DMSO-*d*6.
Figure S19: The COSY spectrum of compound **14** in DMSO-*d*6.
Figure S20: The HSQC spectrum of compound **14** in DMSO-*d*6.
Figure S21: The HMBC spectrum of compound **14** in DMSO-*d*6.
Figure S22: Mass spectrum in negative ion mode (fragmentor = 180 V) of compound **16.**
Figure S23: ^{13}C NMR spectrum (100 MHz) of compound **16** in DMSO-*d*6.
Figure S24: The UV spectrum of compound **16.**
Figure S25: ^{1}H NMR spectrum (400 MHz) of compound **16** in DMSO-*d*6.
Figure S26: The COSY spectrum of compound **16** in DMSO-*d*6.
Figure S27: The HMQC spectrum of compound **16** in DMSO-*d*6.
Figure S28: The HMBC spectrum of compound **16** in DMSO-*d*6.
Figure S29: The UV spectrum of compound **18.**
Figure S30: ^{13}C NMR spectrum (100 MHz) of compound **18** in DMSO-*d*6.
Figure S31: Mass spectrum in negative ion mode (fragmentor = 180 V) of compound **18.**
Figure S32: ^{1}H NMR spectrum (400 MHz) of compound **18** in DMSO-*d*6.
Figure S33: The COSY spectrum of compound **18** in DMSO-*d*6.
Figure S34: The HMQC spectrum of compound **18** in DMSO-*d*6.
Figure S35: The HMBC spectrum of compound **18** in DMSO-*d*6.
Figure S36: The DEPT spectrum of compound **16** in DMSO-*d*6.
Figure S37: The UV chromatogram of extract **HP1** obtained by LC-PDA-HRMS.
Figure S38: The UV with crucial MS chromatogram of extract **HP2** obtained by LC-PDA-HRMS.
Figure S39: The UV with crucial MS chromatogram of extract **HP3** obtained by LC-PDA-HRMS.
Figure S40: The UV chromatogram of fraction **HP4** obtained by LC-PDA-HRMS.
Figure S41: The UV with crucial MS chromatograms of fraction **HP5** obtained by LC-PDA-HRMS.
Figure S42: The UV chromatogram of extract **HP6** obtained by LC-PDA-HRMS.
Figure S43: The UV chromatogram of extract **HP7** obtained by LC-PDA-HRMS.
Figure S44: The UV with crucial MS chromatograms of fraction **HP8** obtained by LC-PDA-HRMS.

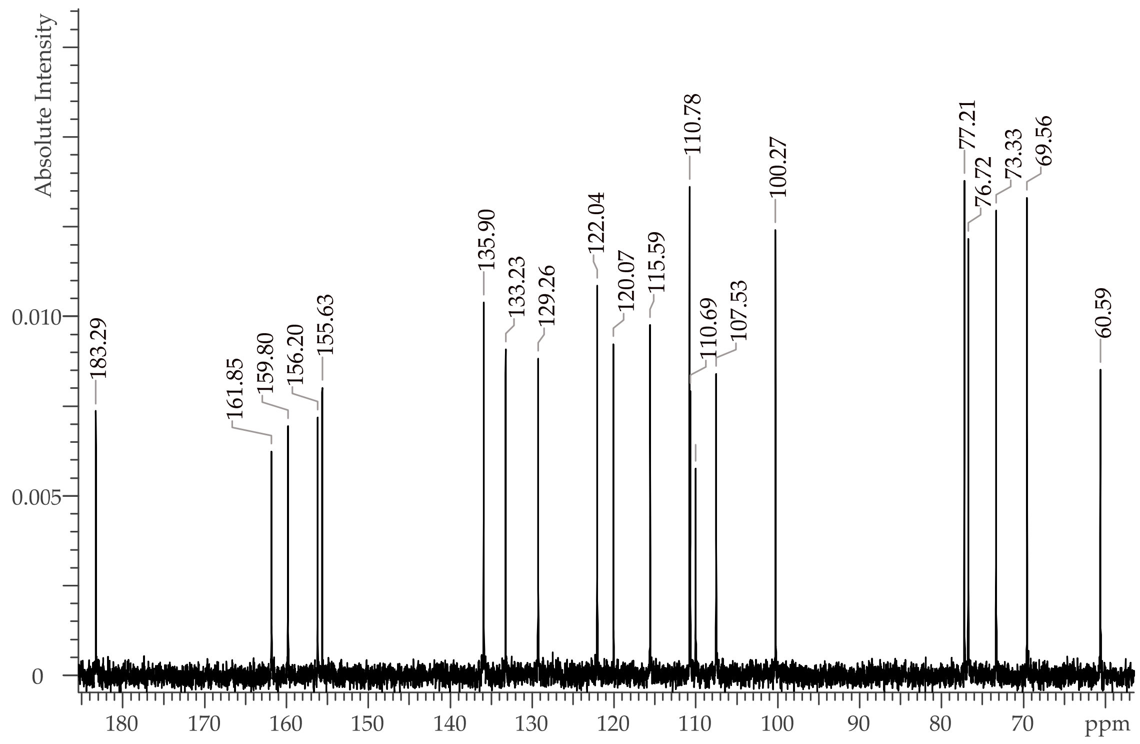

Figure S1: ^{13}C NMR spectrum (100 MHz) of compound **9** in DMSO-*d6*.

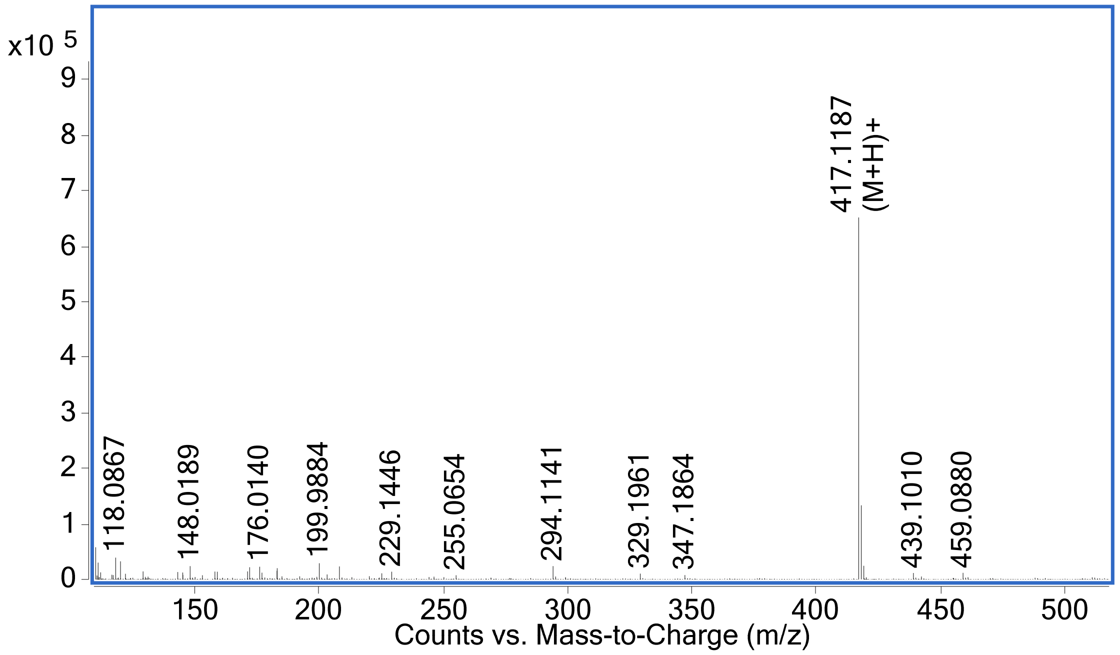

Figure S2: Mass spectrum in positive ion mode (fragmentor = 180 V) of compound **9.**

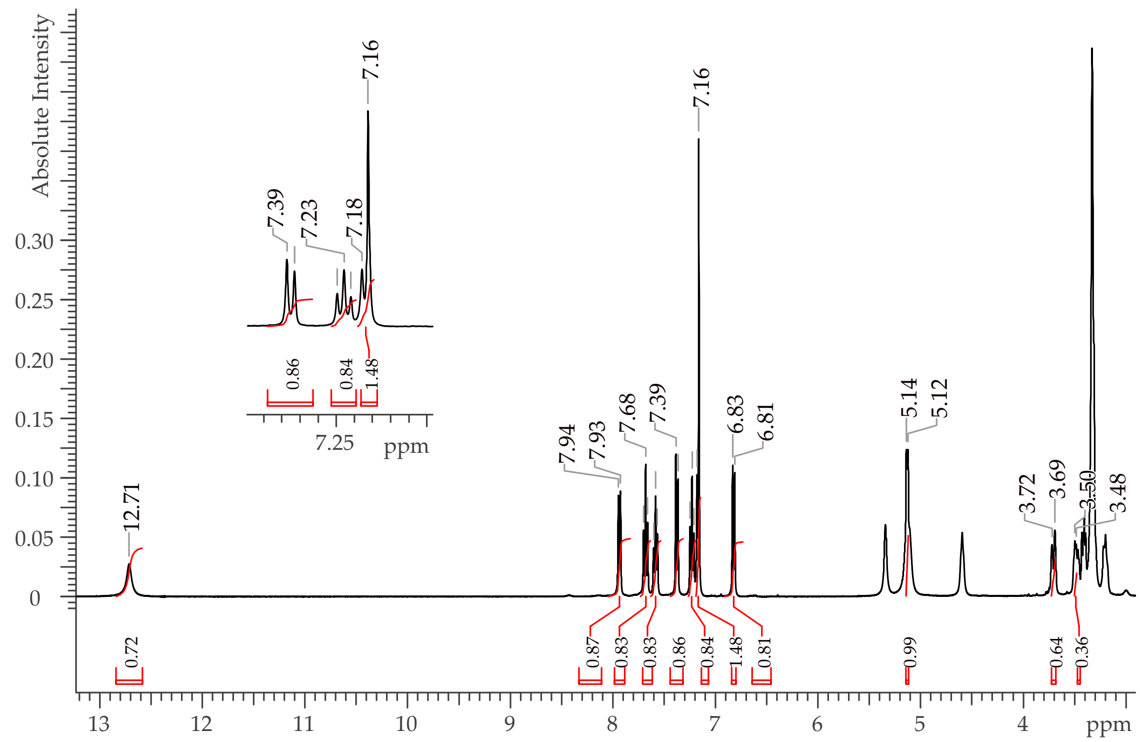

Figure S3: ^{1}H NMR spectrum (400 MHz) of compound **9** in DMSO-*d6*.

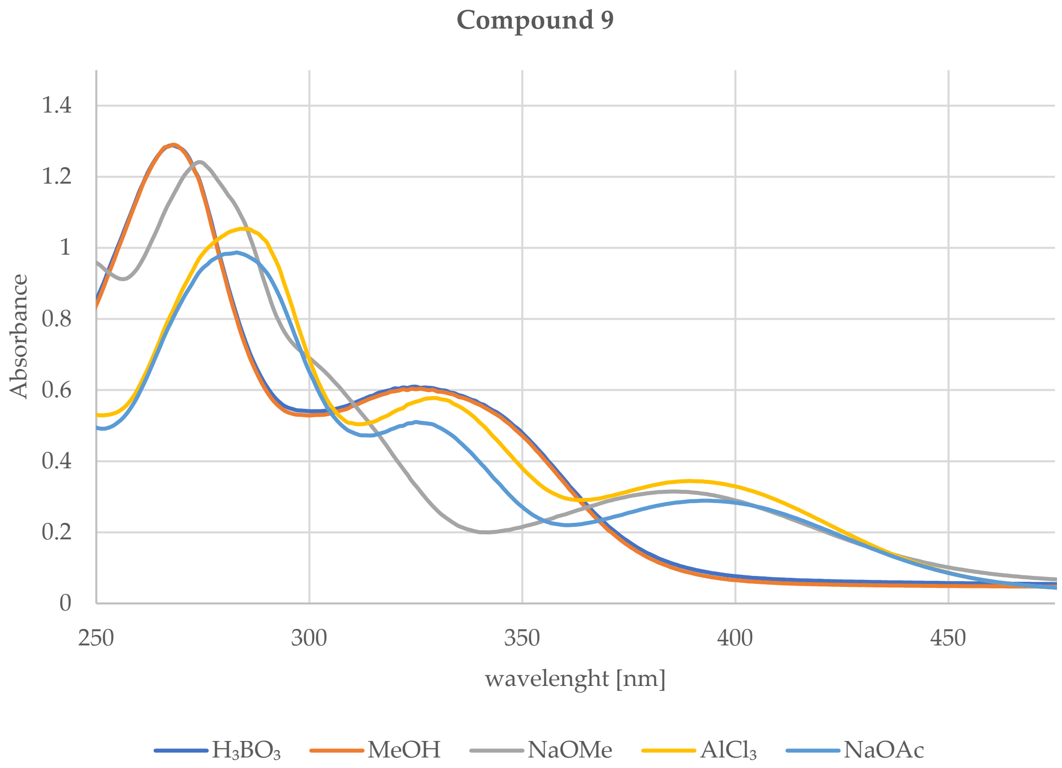

Figure S4: The UV spectrum of compound **9.**

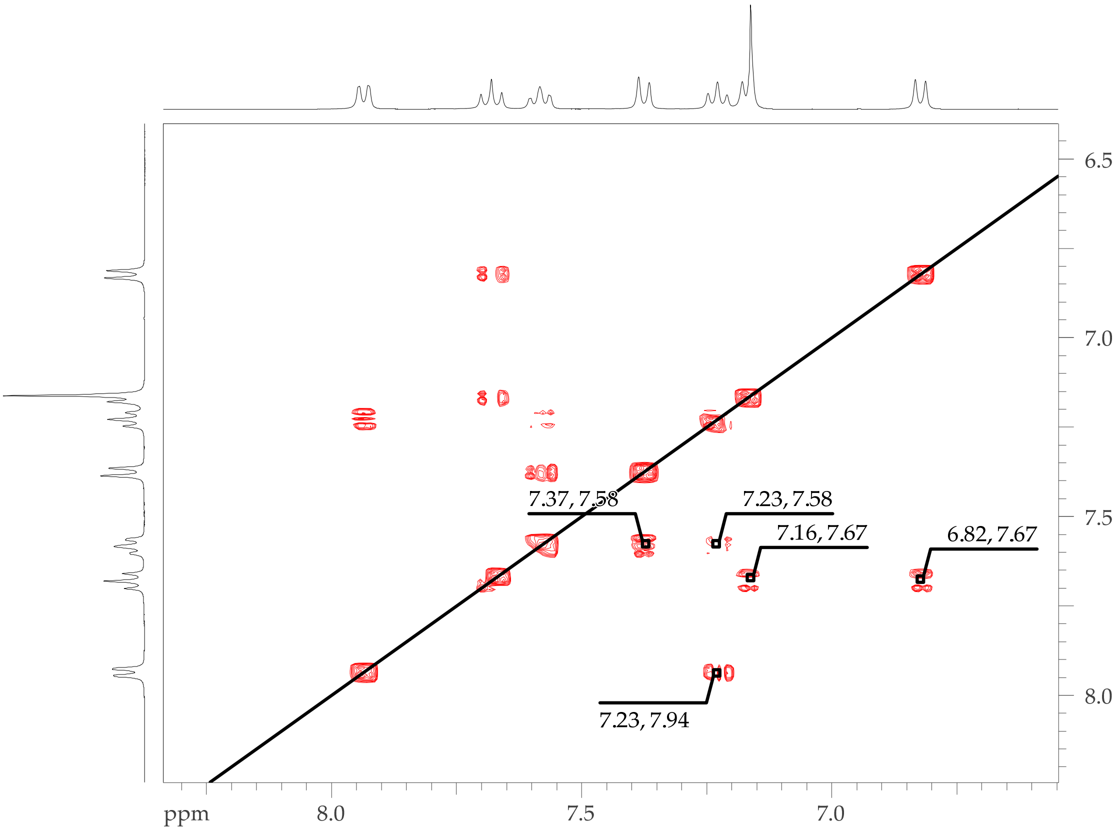

Figure S5: The COSY spectrum of compound **9** in DMSO-*d6*.

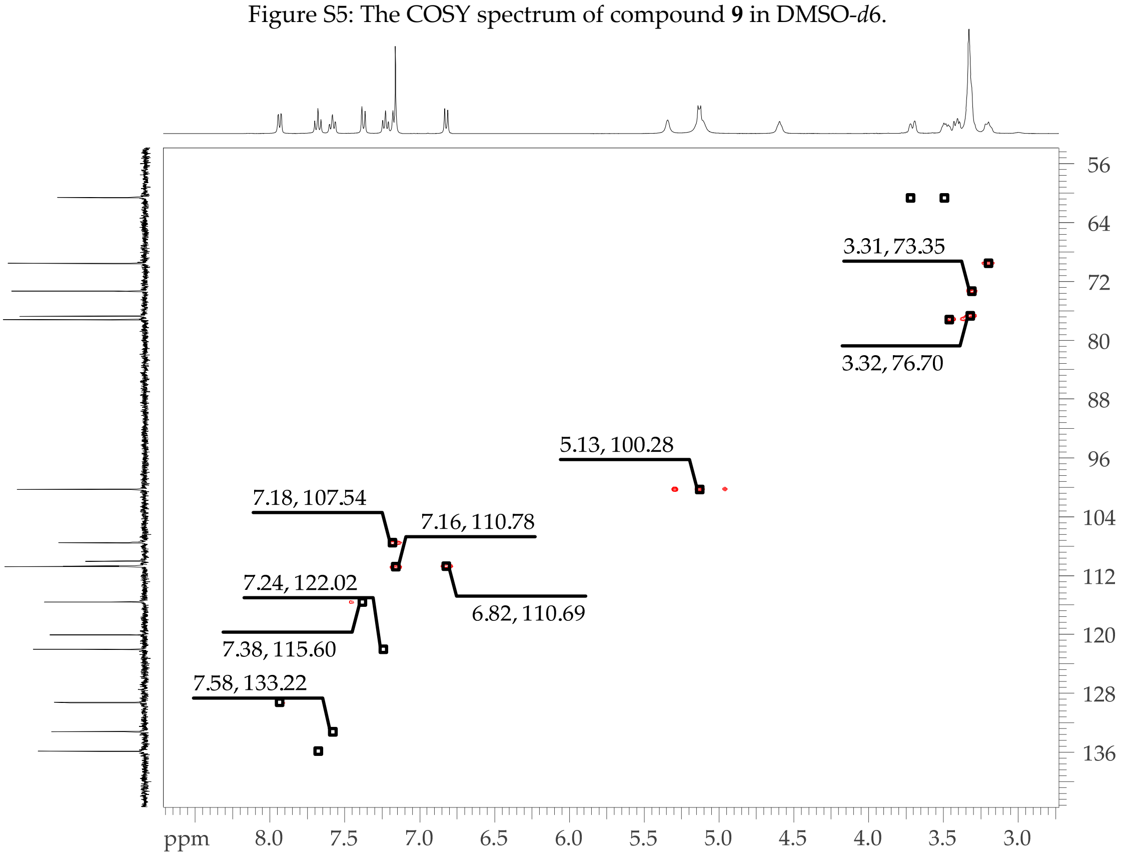

Figure S6: The HSQC spectrum of compound **9** in DMSO-*d6*.

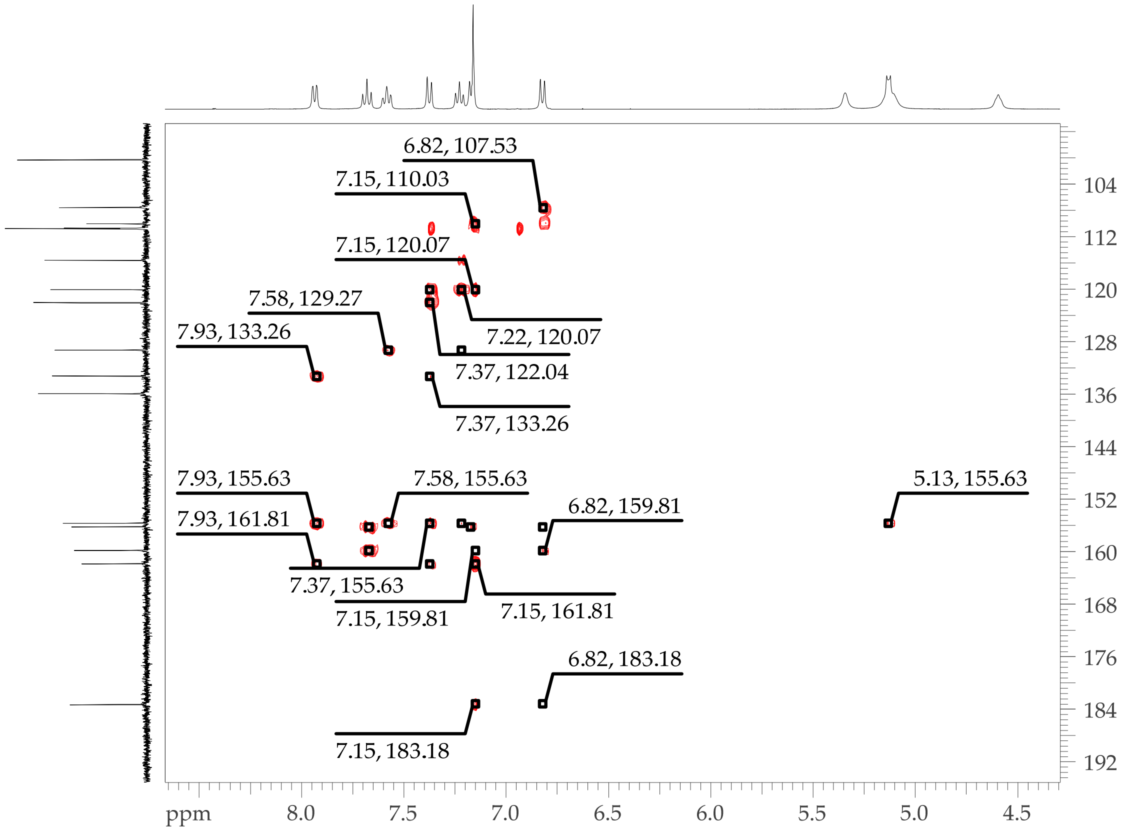

Figure S7: The HMBC spectrum of compound **9** in DMSO-*d6*.

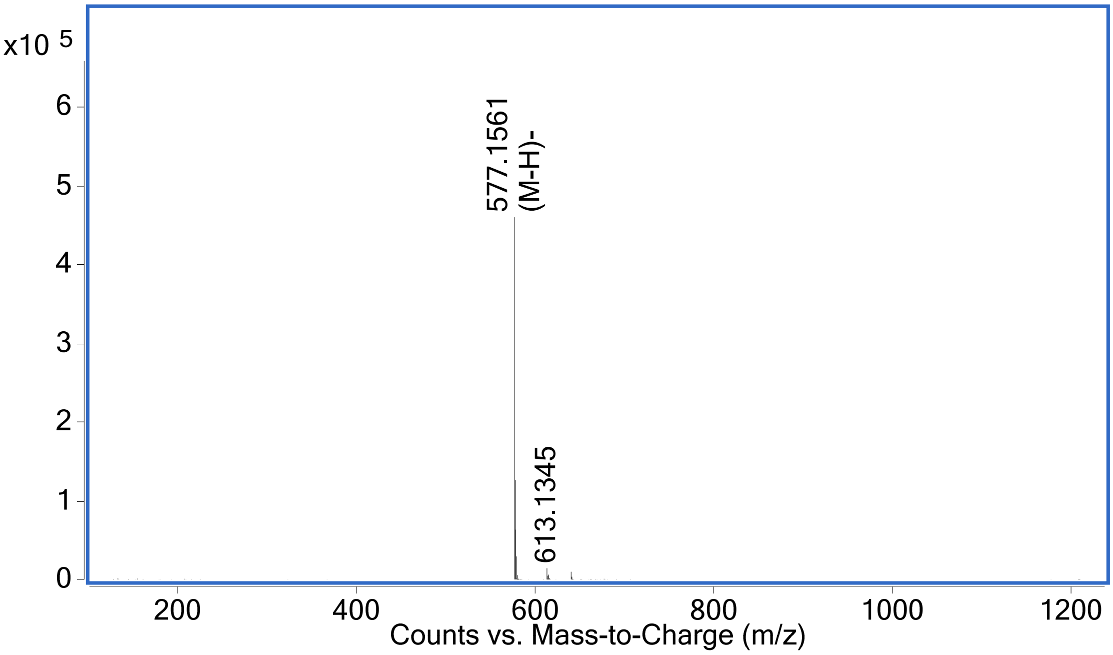

Figure S8: Mass spectrum in negative ion mode (fragmentor = 180 V) of compound **10.**

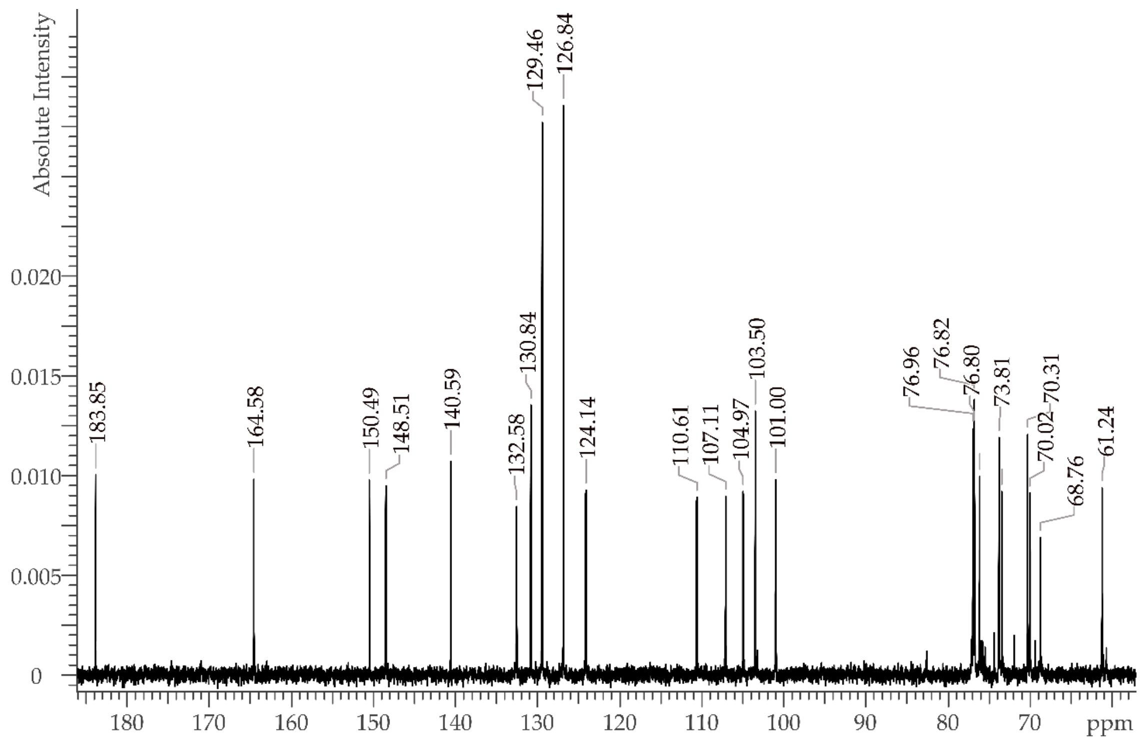

Figure S9: ^{13}C NMR spectrum (100 MHz) of compound **10** in DMSO-*d*6.

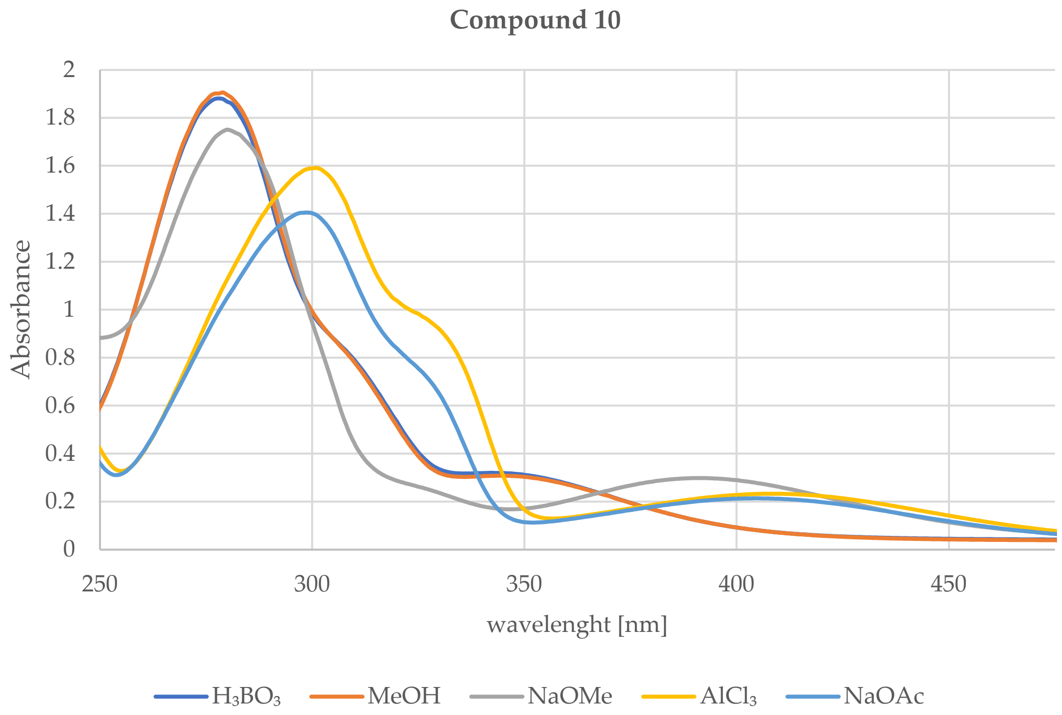

Figure S10: The UV spectrum of compound **10.**

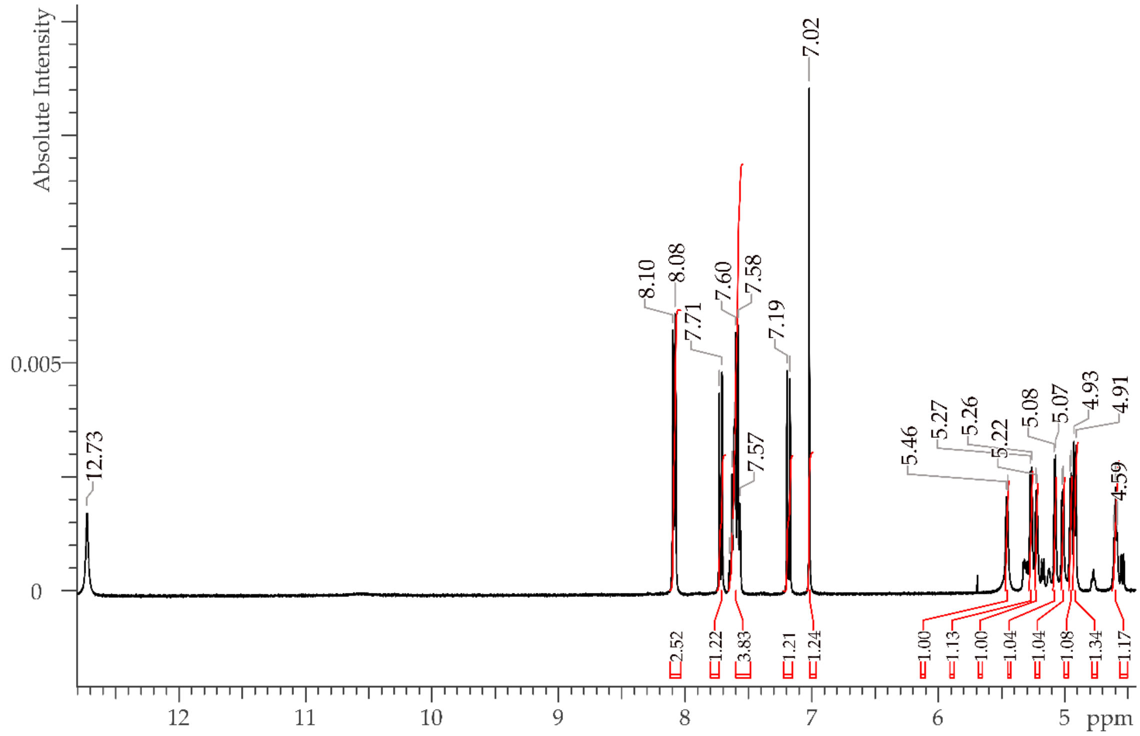

Figure S11: ^{1}H NMR spectrum (400 MHz) of compound **10** in DMSO-*d6*.

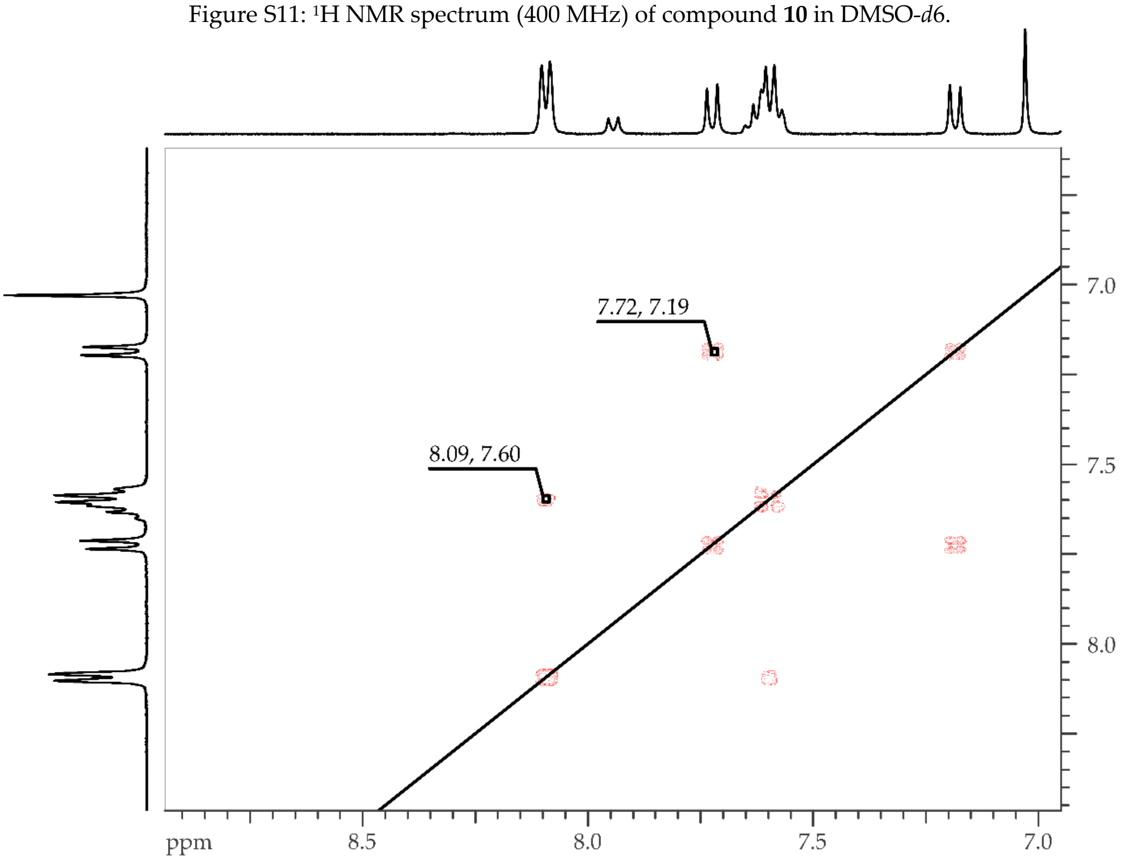

Figure S12: The COSY spectrum of compound **10** in DMSO-*d6*.

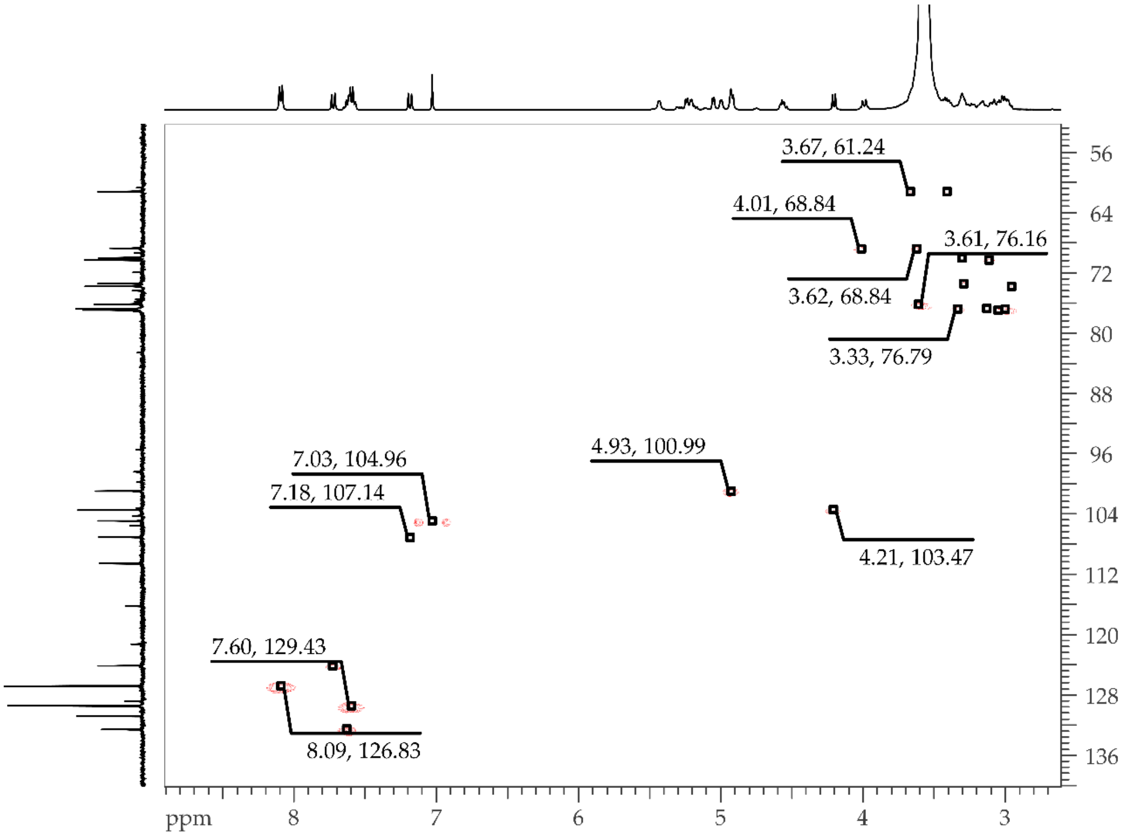

Figure S13: The HMQC spectrum of compound **10** in DMSO-*d*6.

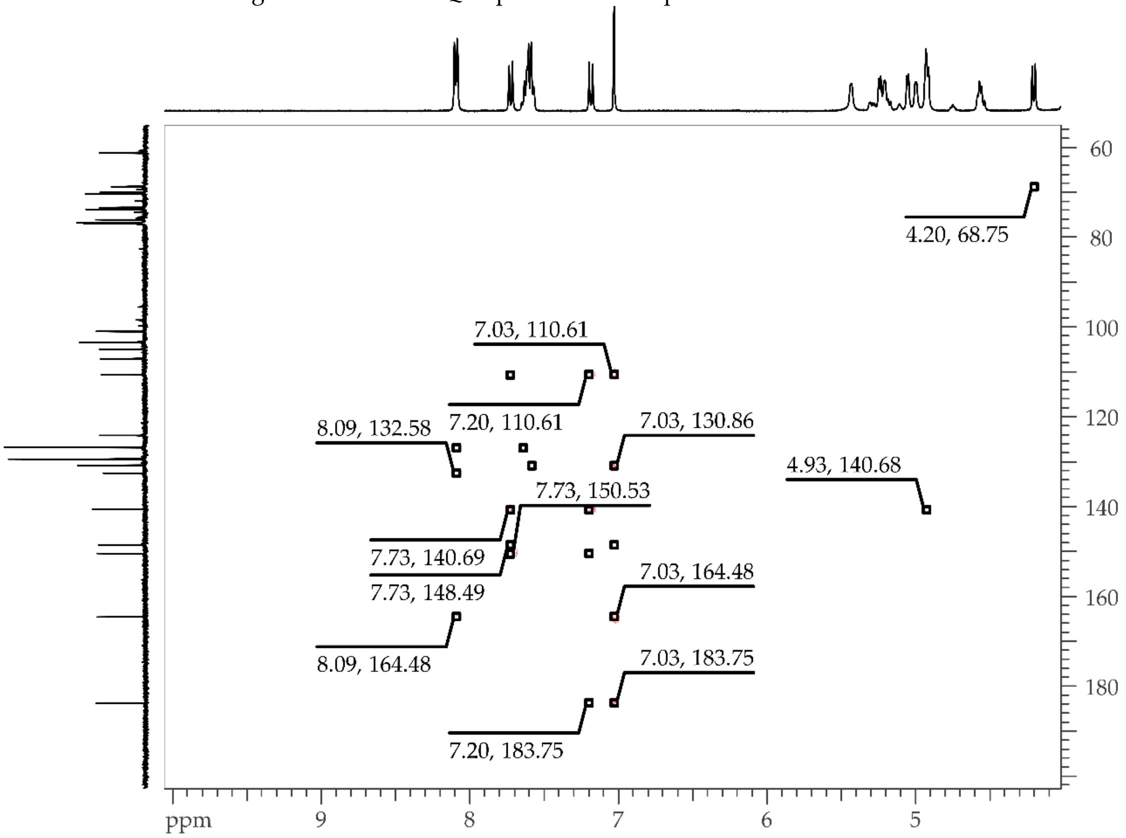

Figure S14: The HMBC spectrum of compound **10** in DMSO-*d*6.

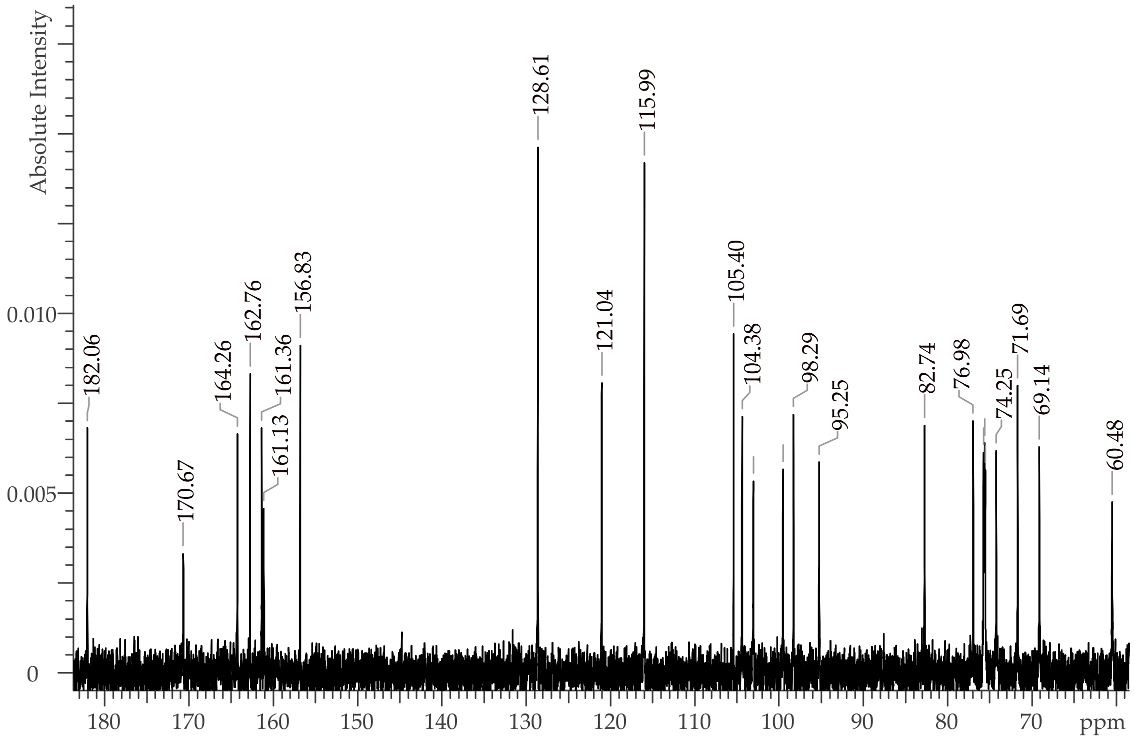

Figure S15: ^{13}C NMR spectrum (100 MHz) of compound **14** in DMSO-*d6*.

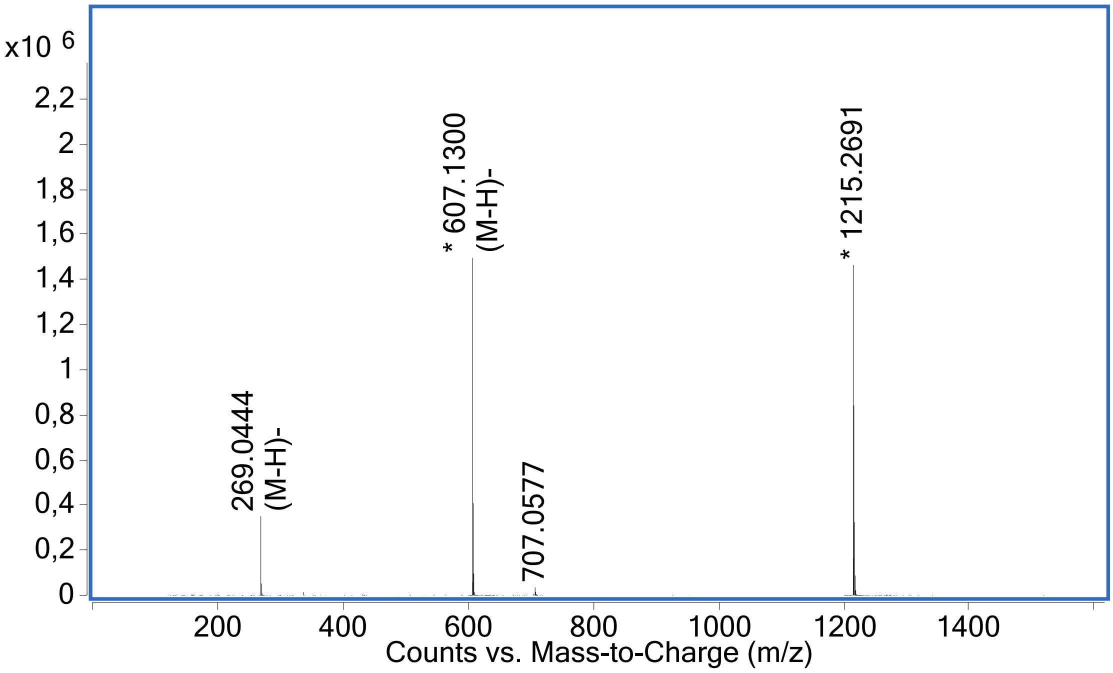

Figure S16: Mass spectrum in negative ion mode (fragmentor = 180 V) of compound **14.**

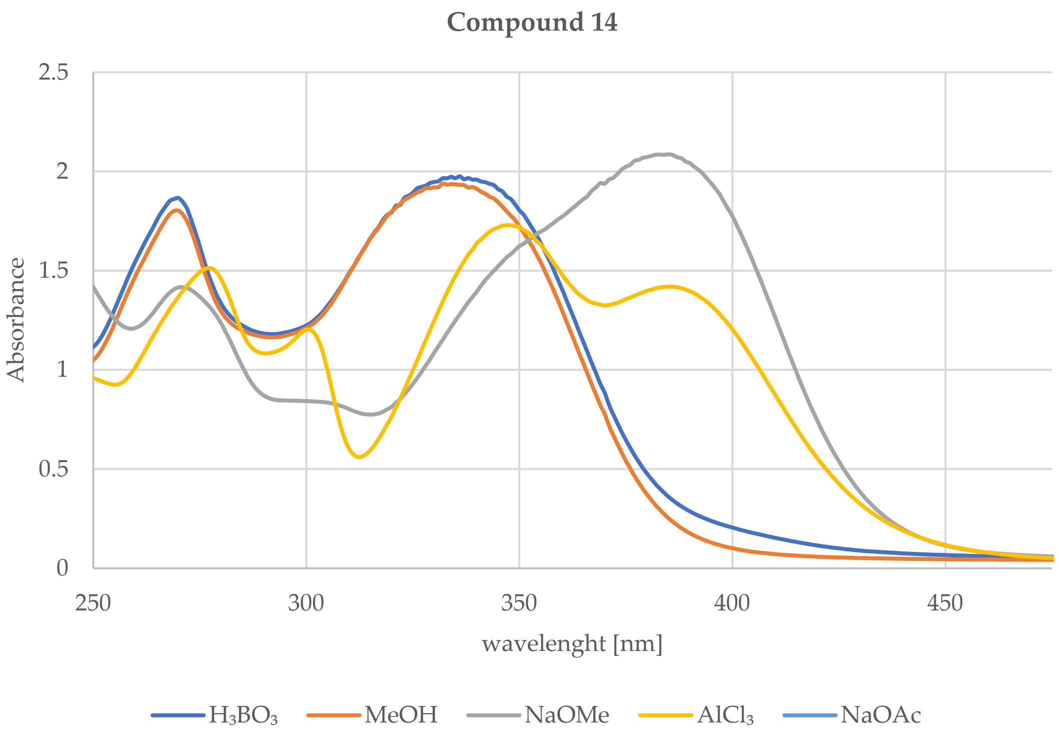

Figure S17: The UV spectrum of compound **14.**

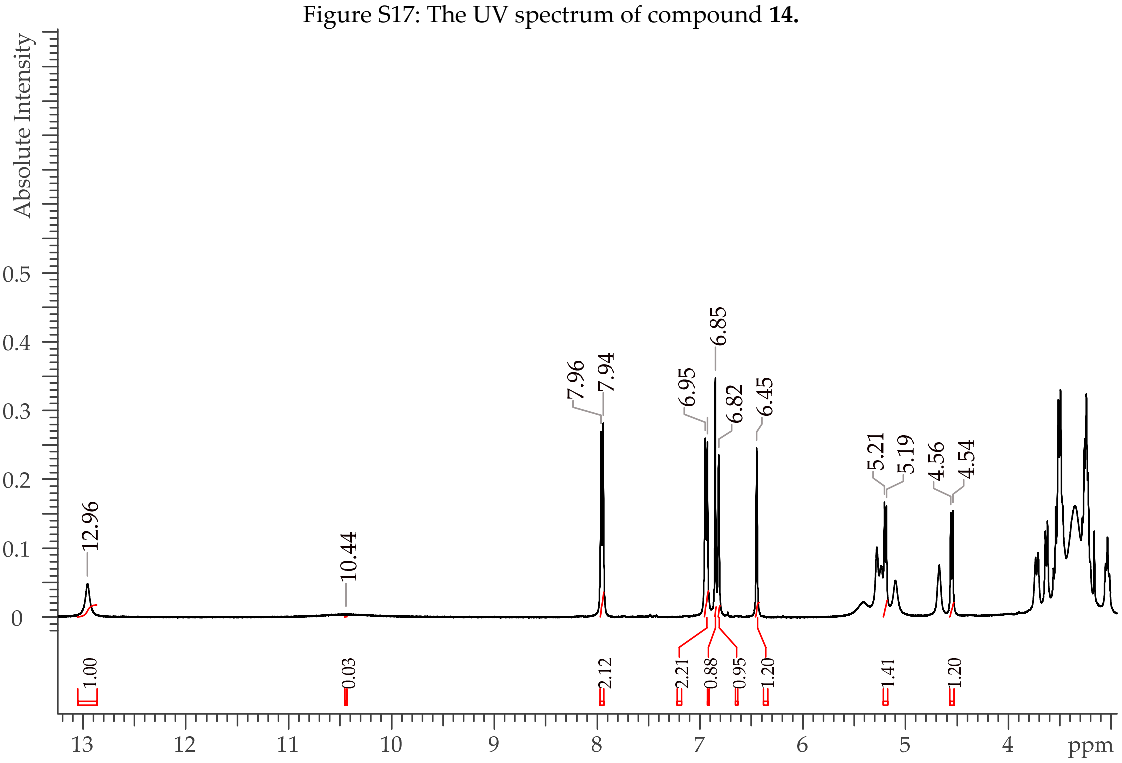

Figure S18: ^{1}H NMR spectrum (400 MHz) of compound **14** in DMSO-*d6*.

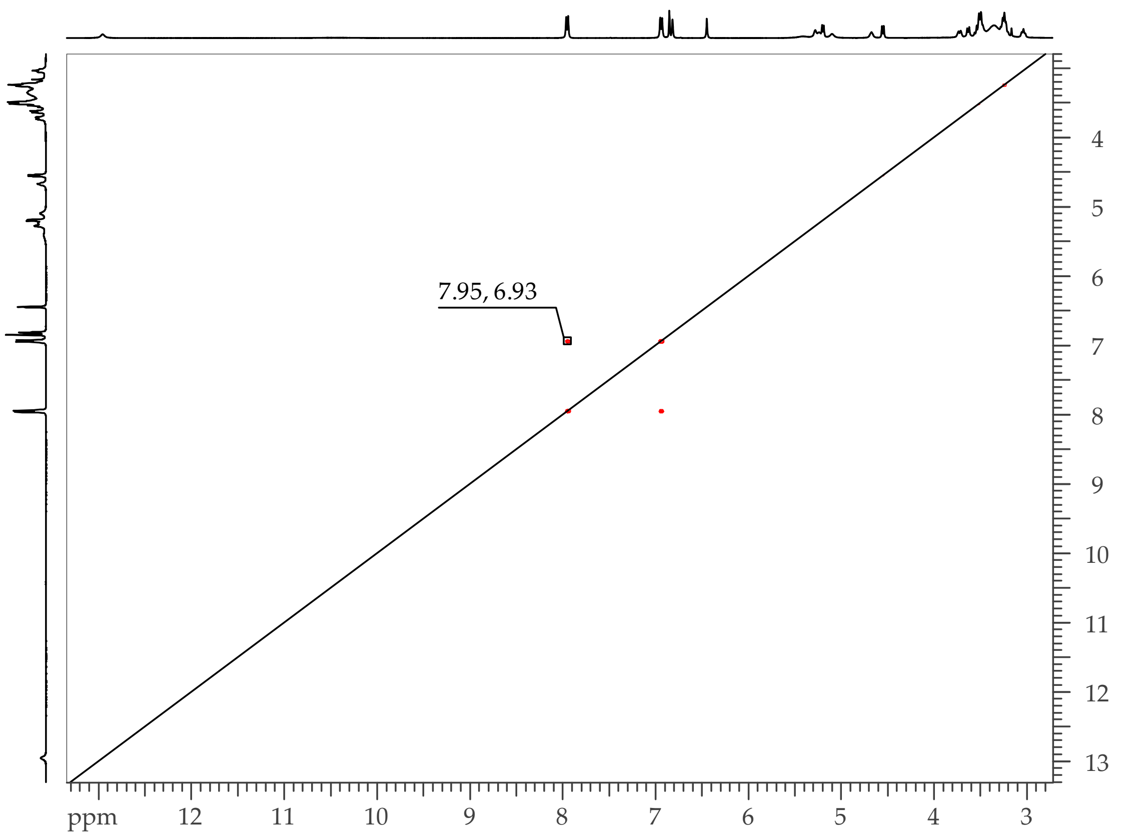

Figure S19: The COSY spectrum of compound **14** in DMSO-*d6*.

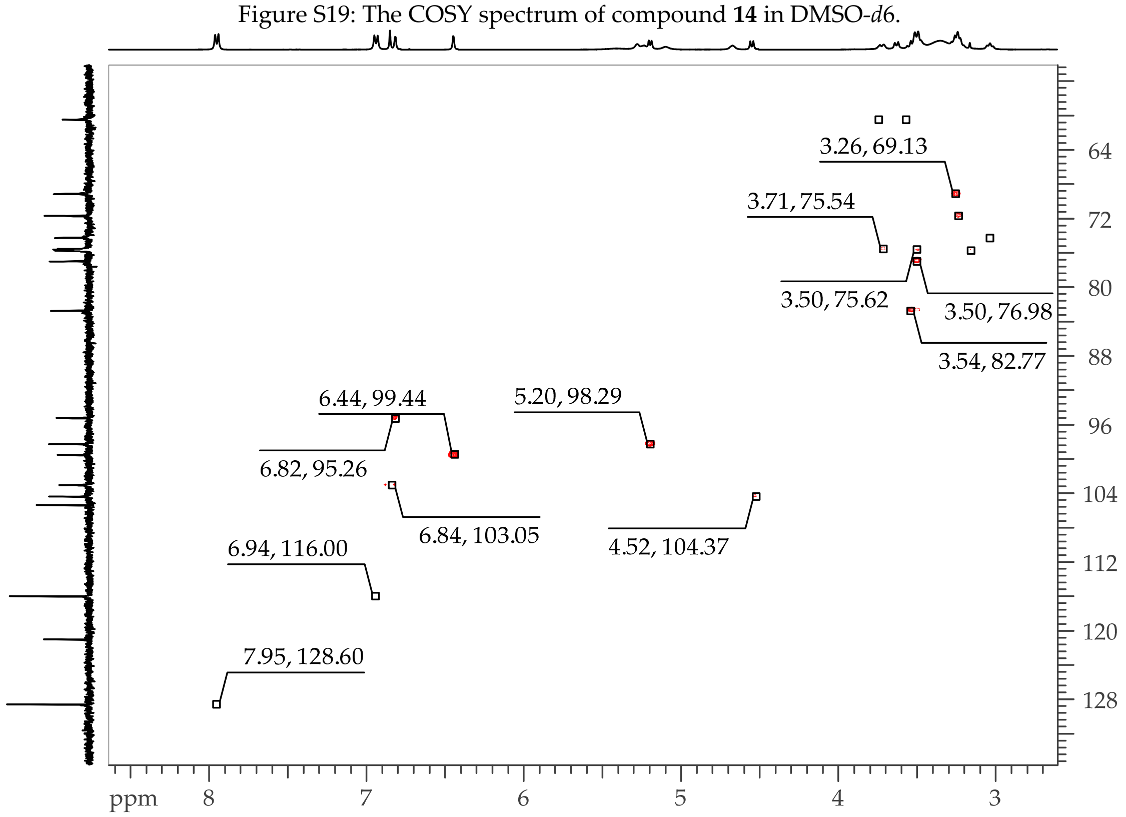

Figure S20: The HSQC spectrum of compound **14** in DMSO-*d6*.

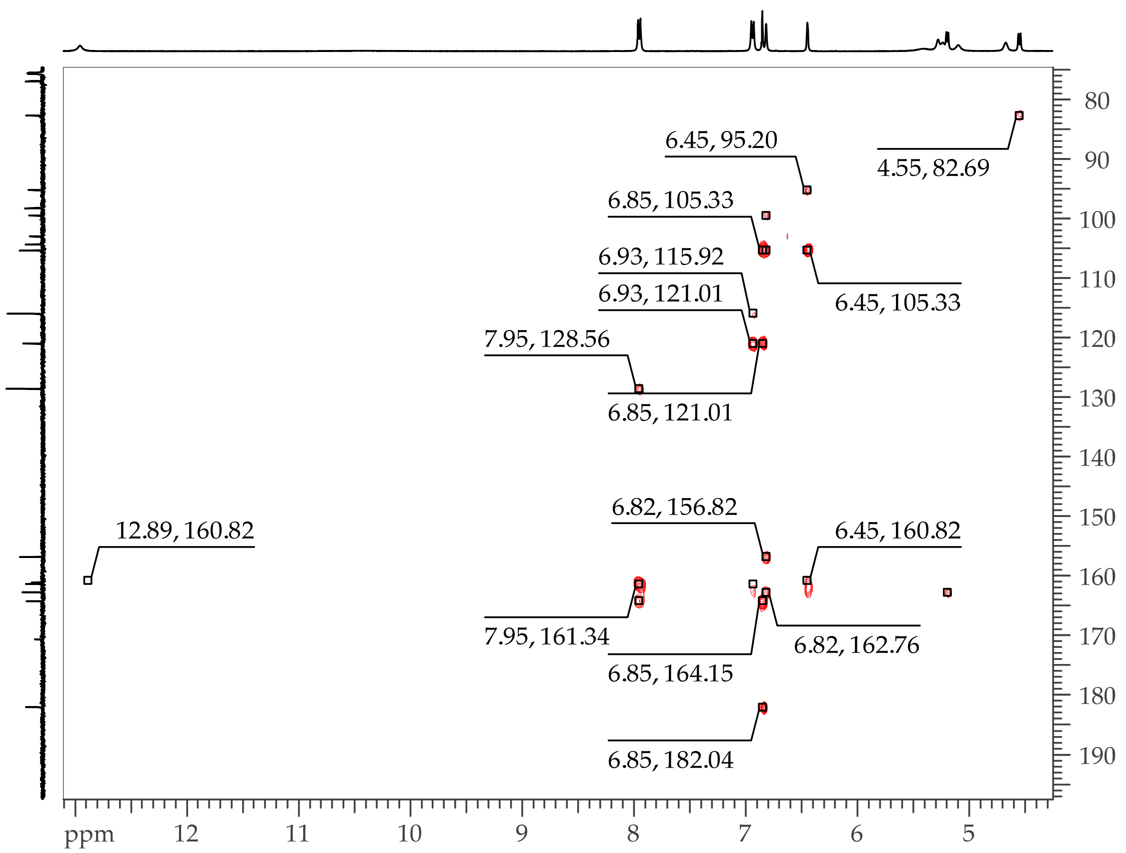

Figure S21: The HMBC spectrum of compound **14** in DMSO-*d6*.

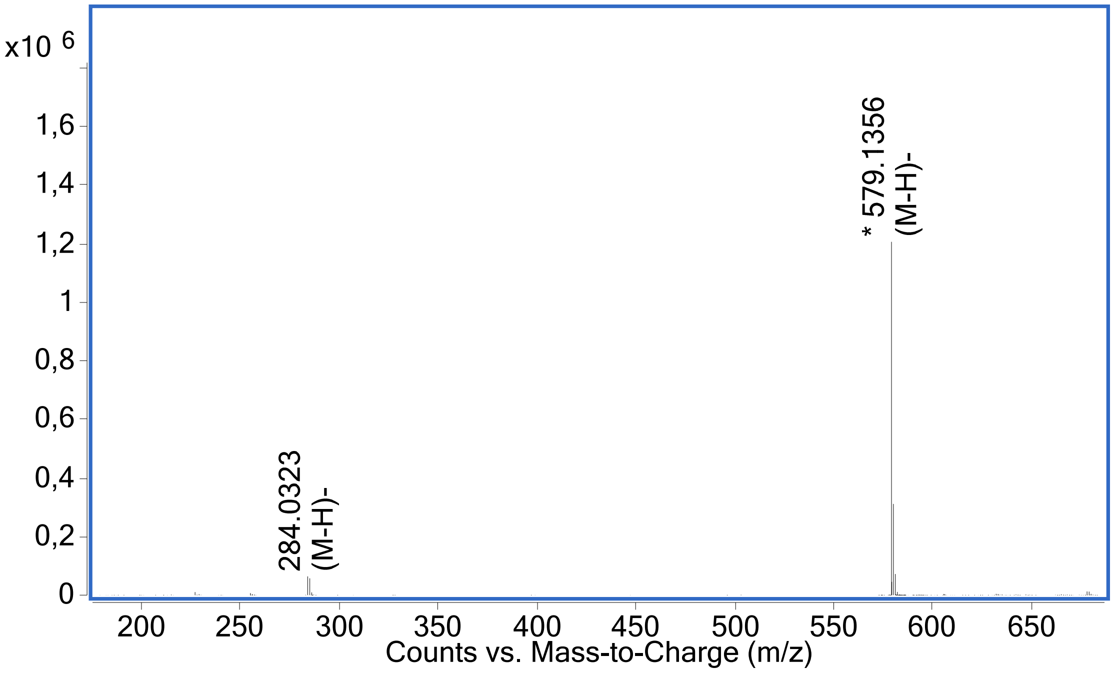

Figure S22: Mass spectrum in negative ion mode (fragmentor = 180 V) of compound **16.**

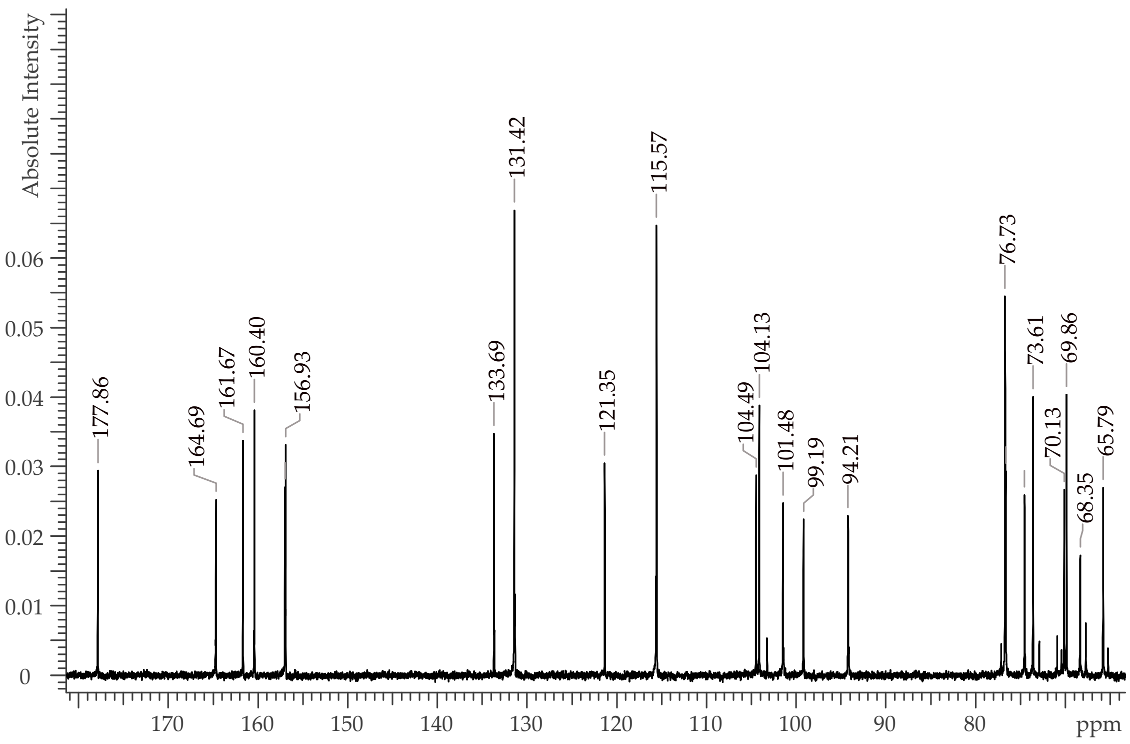

Figure S23: ^{13}C NMR spectrum (100 MHz) of compound **16** in DMSO-*d*6.

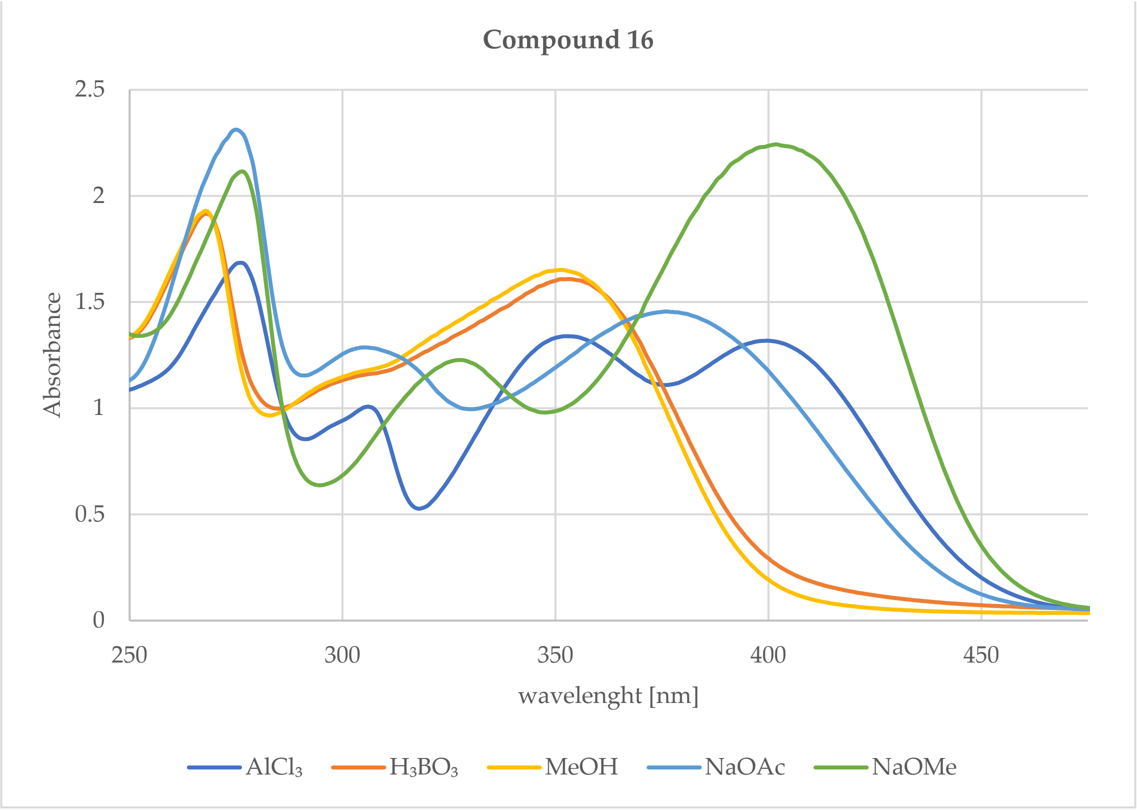

Figure S24: The UV spectrum of compound **16.**

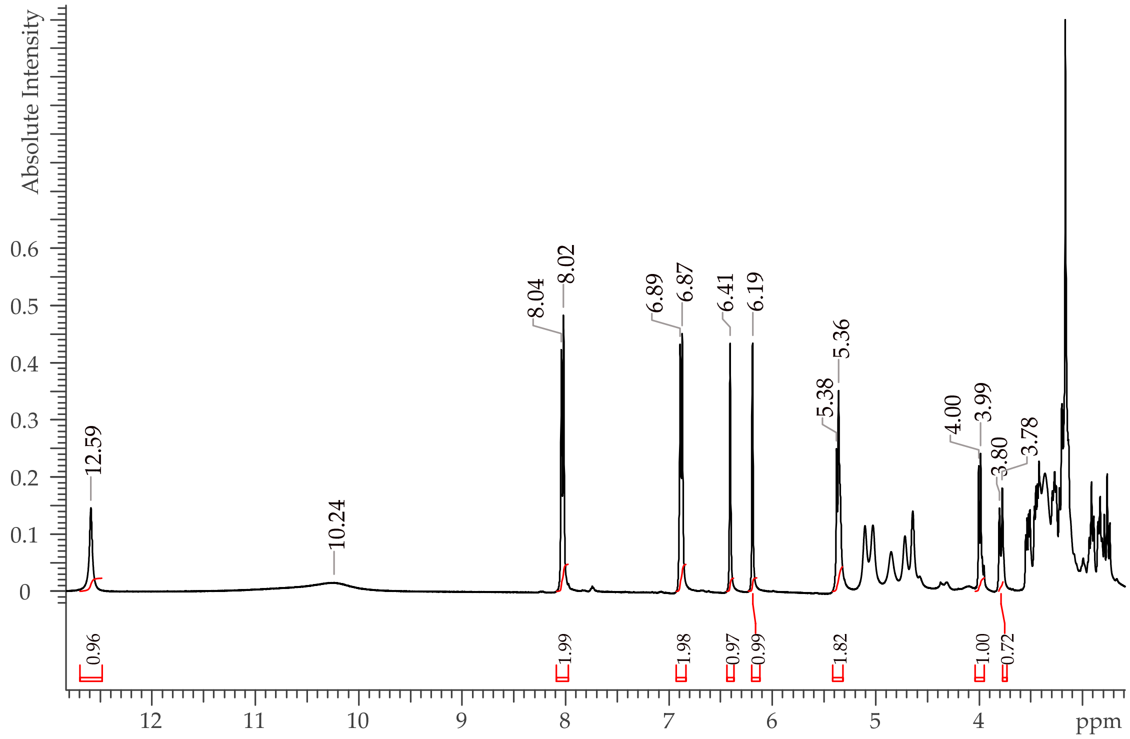

Figure S25: ¹H NMR spectrum (400 MHz) of compound **16** in DMSO-*d*6.

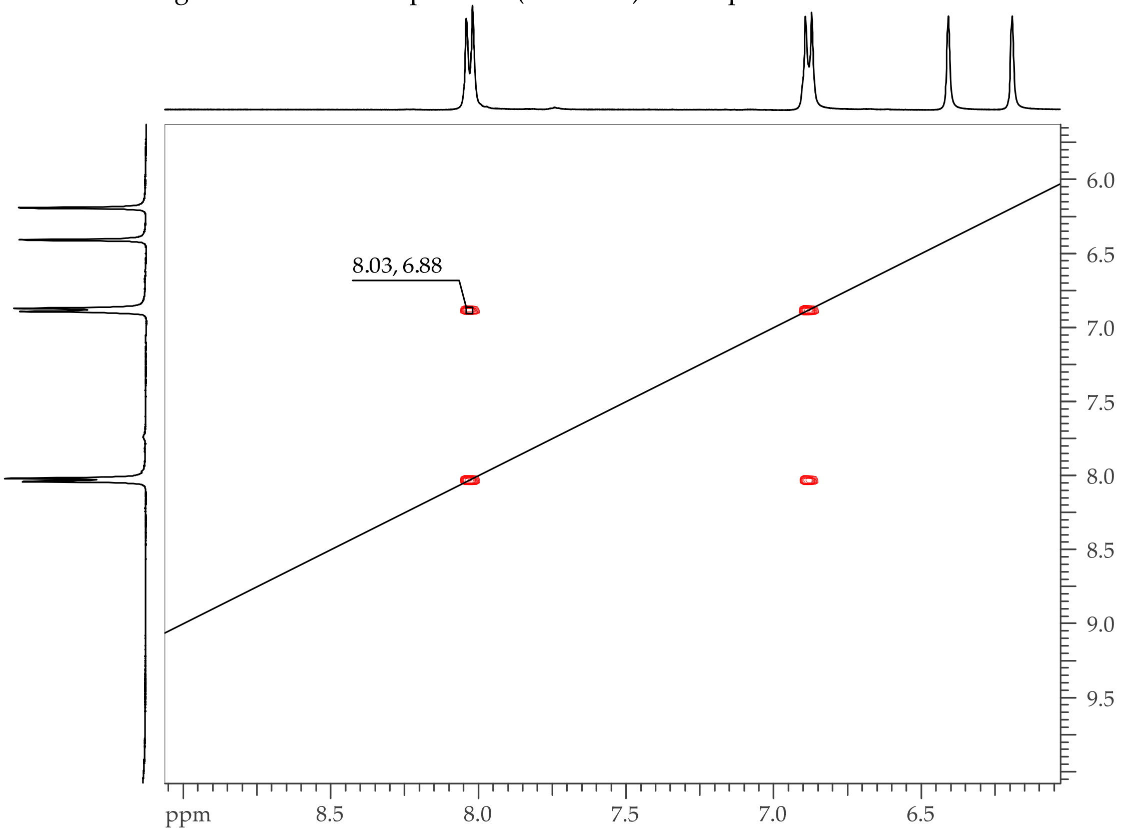

Figure S26: The COSY spectrum of compound **16** in DMSO-*d*6.

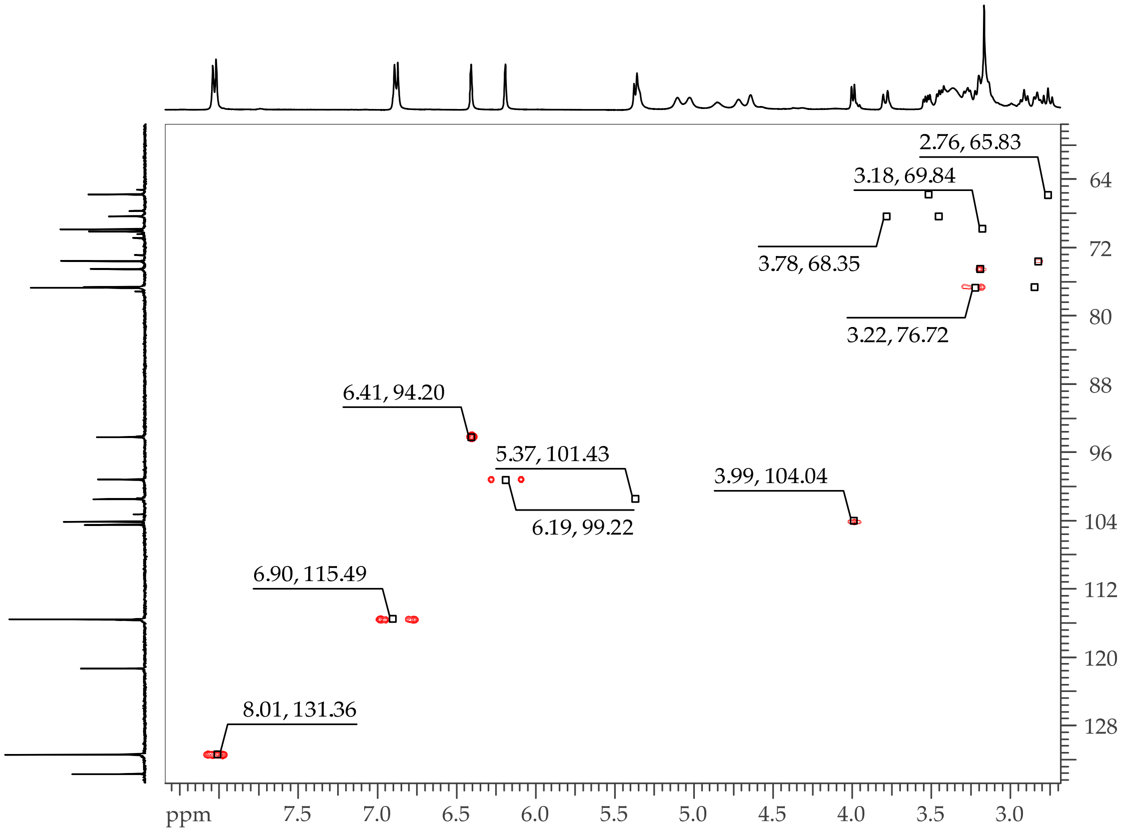

Figure S27: The HMQC spectrum of compound **16** in DMSO-*d6*.

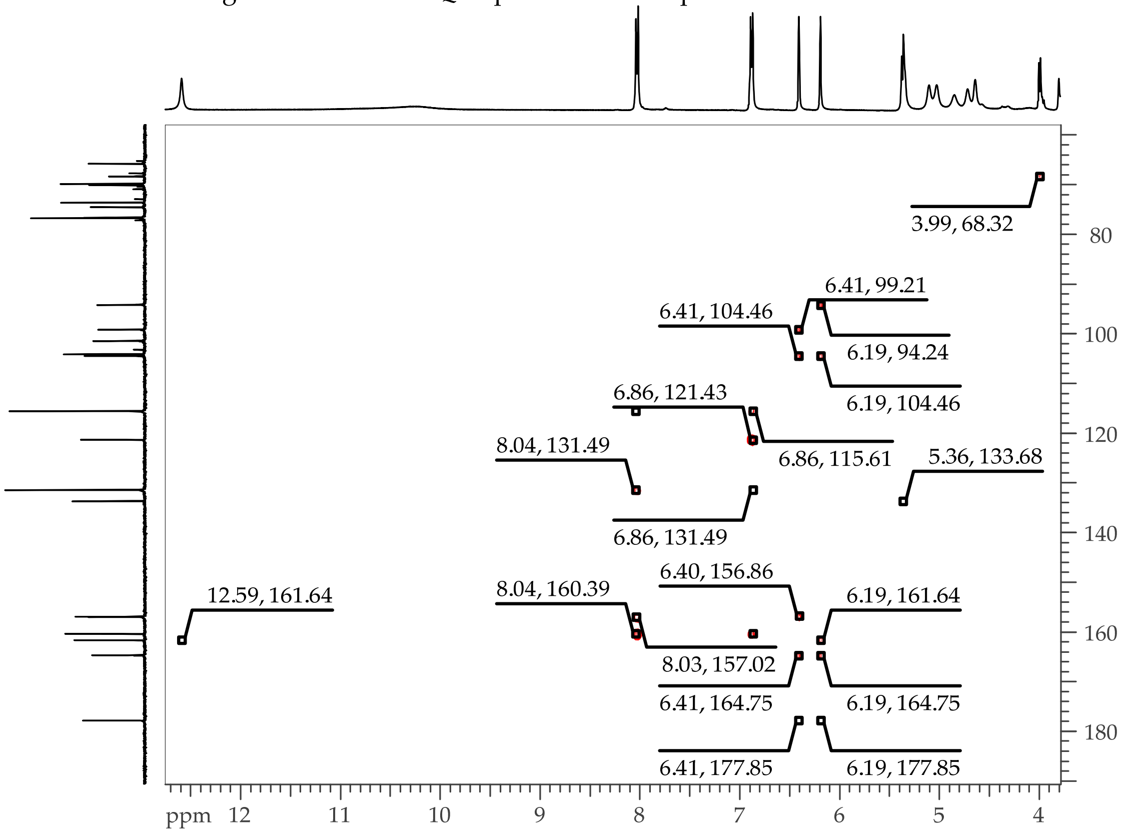

Figure S28: The HMBC spectrum of compound **16** in DMSO-*d6*.

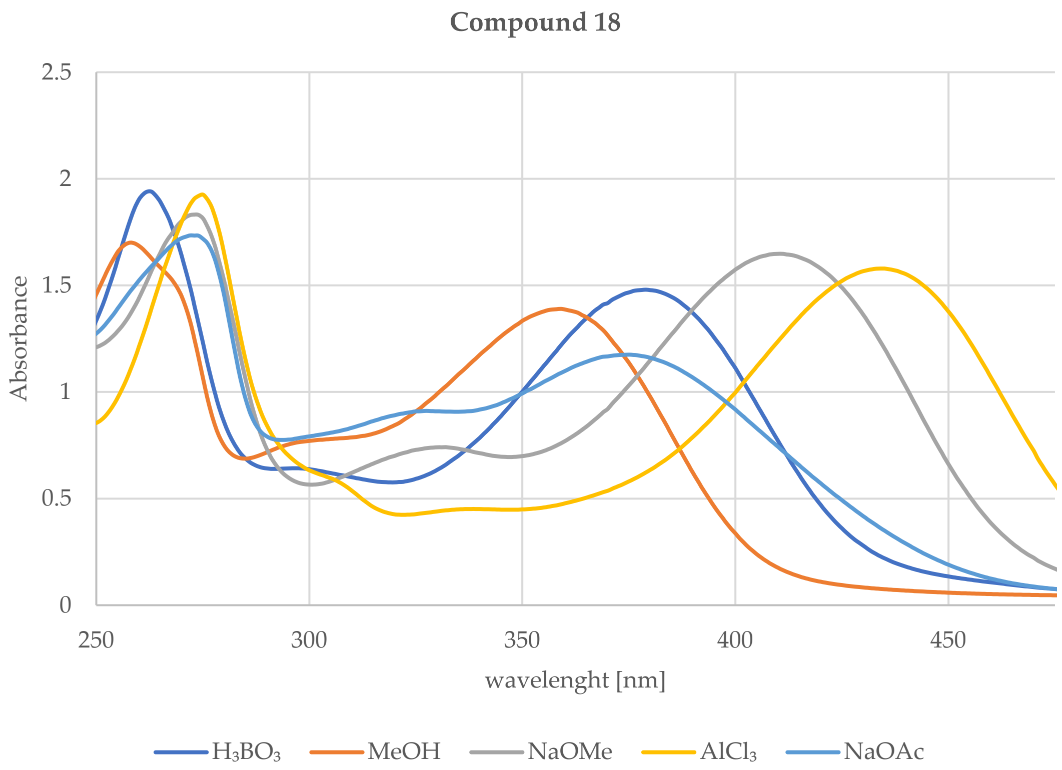

Figure S29: The UV spectrum of compound **18.**

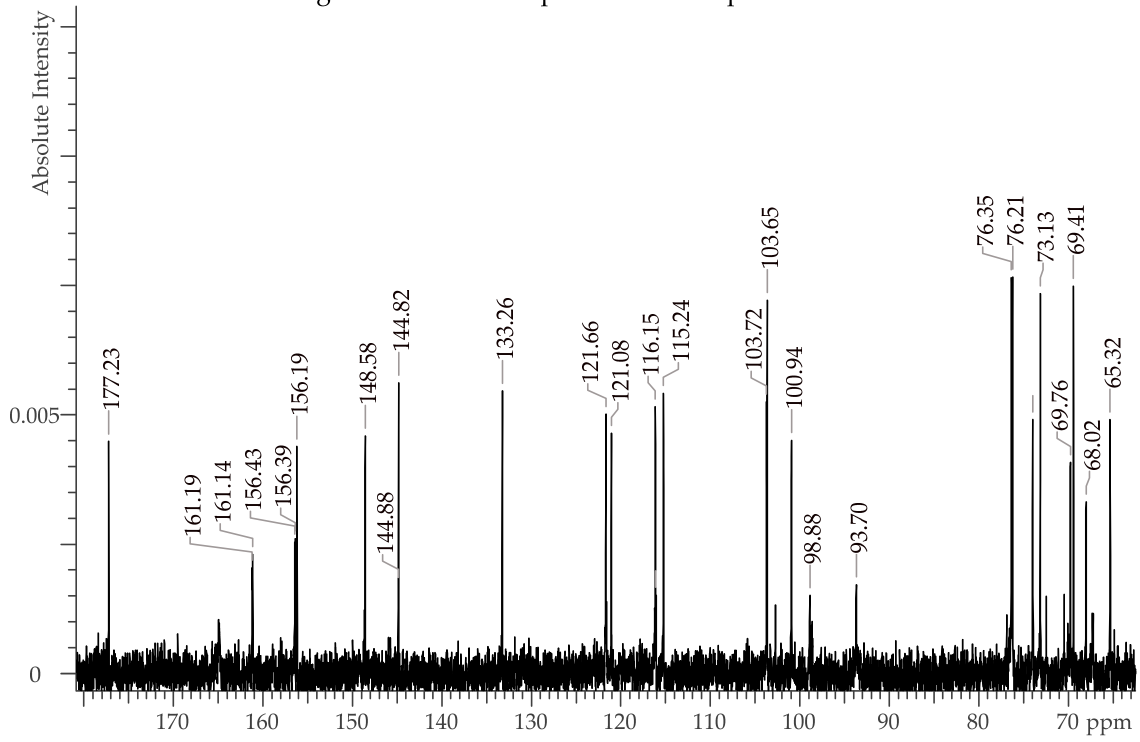

Figure S30: ^{13}C NMR spectrum (100 MHz) of compound **18** in DMSO-*d6*.

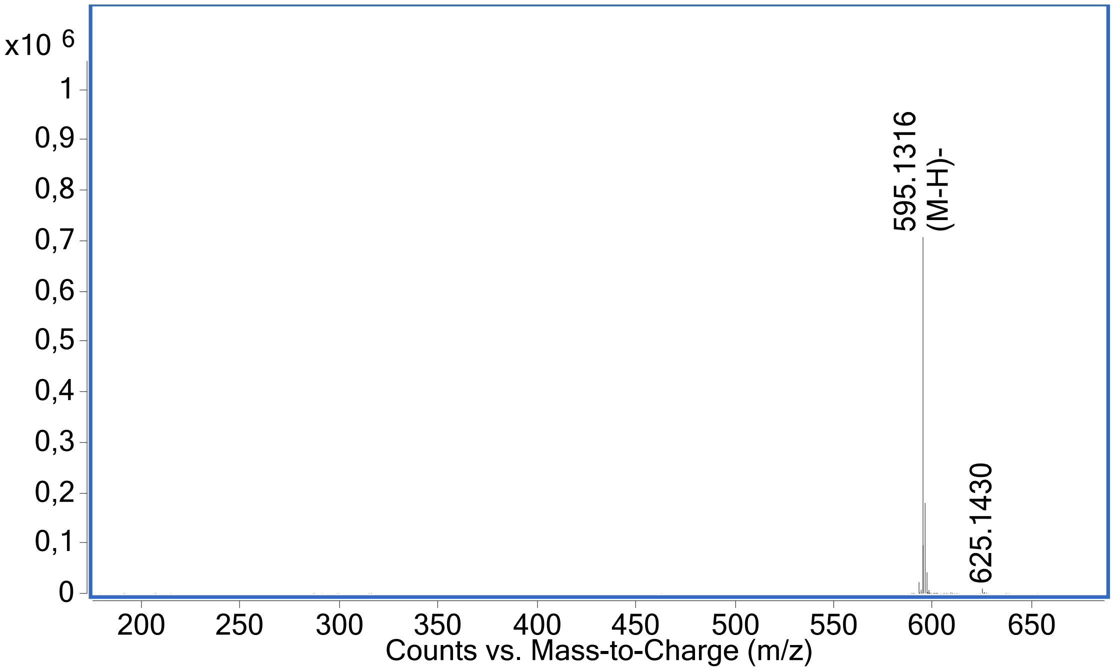

Figure S31: Mass spectrum in negative ion mode (fragmentor = 180 V) of compound **18.**

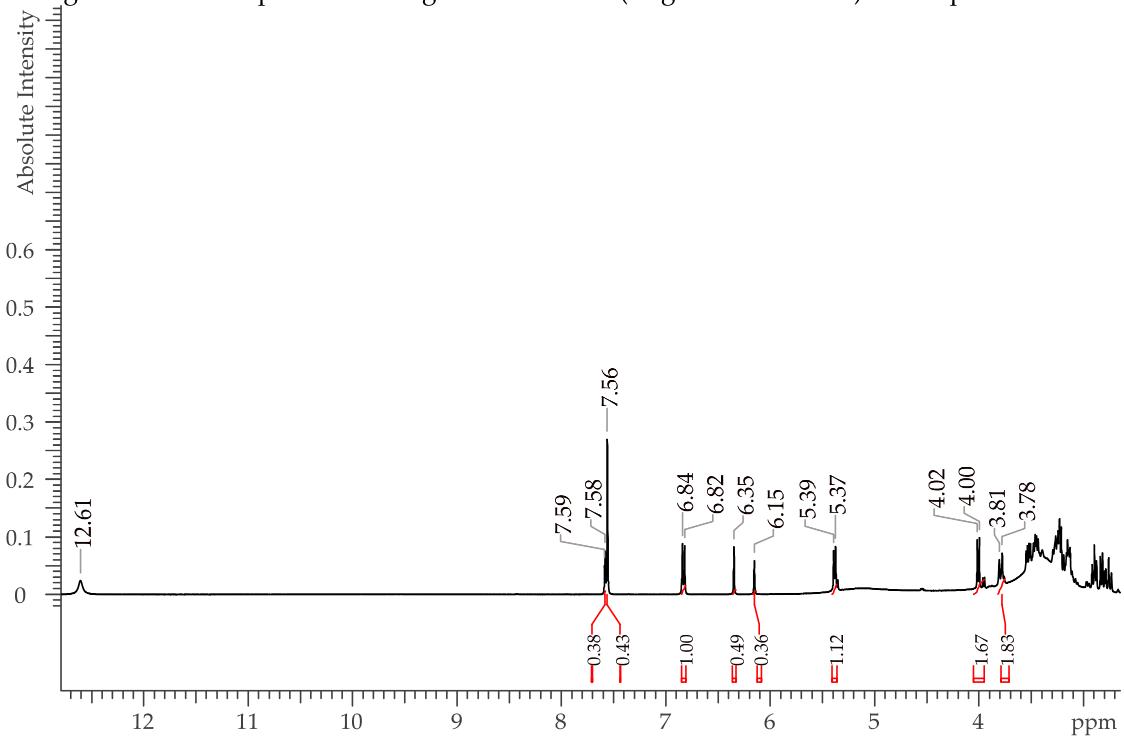

Figure S32: ^{1}H NMR spectrum (400 MHz) of compound **18** in DMSO-*d*6.

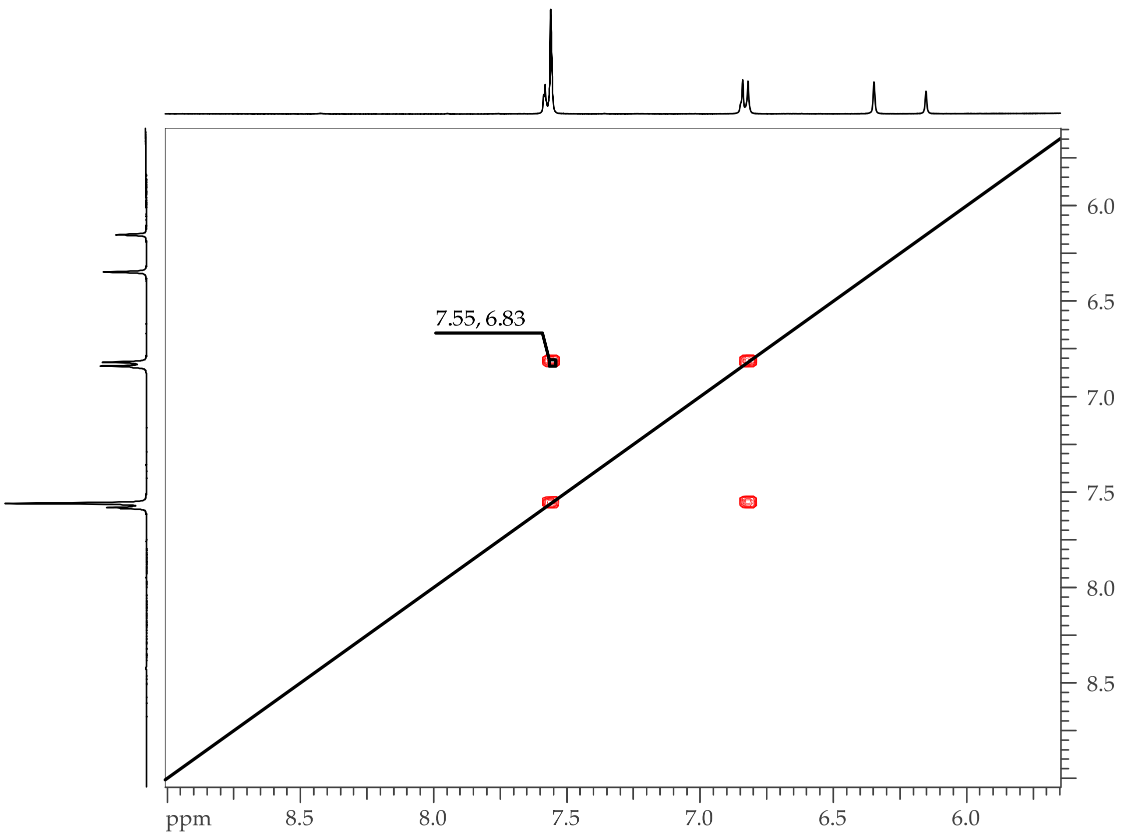

Figure S33: The COSY spectrum of compound **18** in DMSO-*d*6.

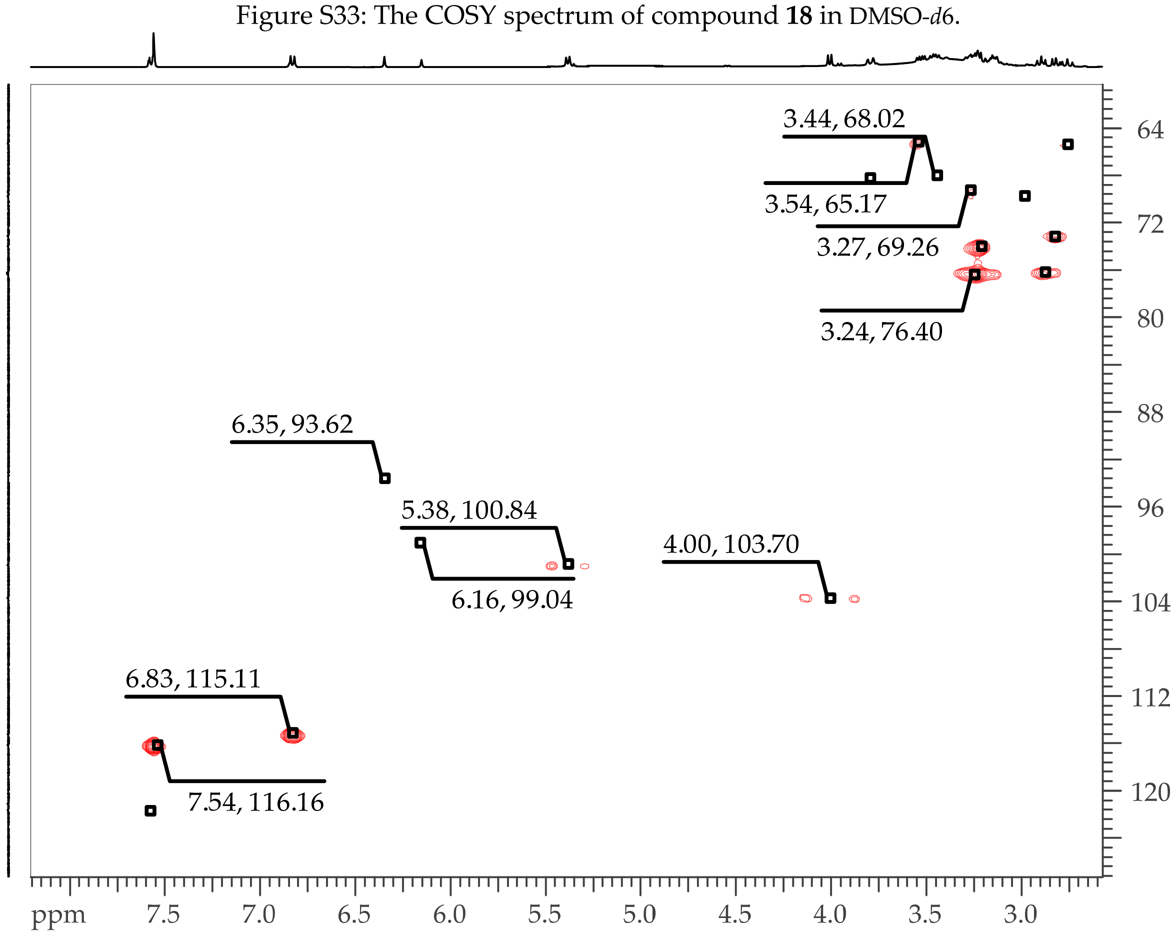

Figure S34: The HMQC spectrum of compound **18** in DMSO-*d*6.

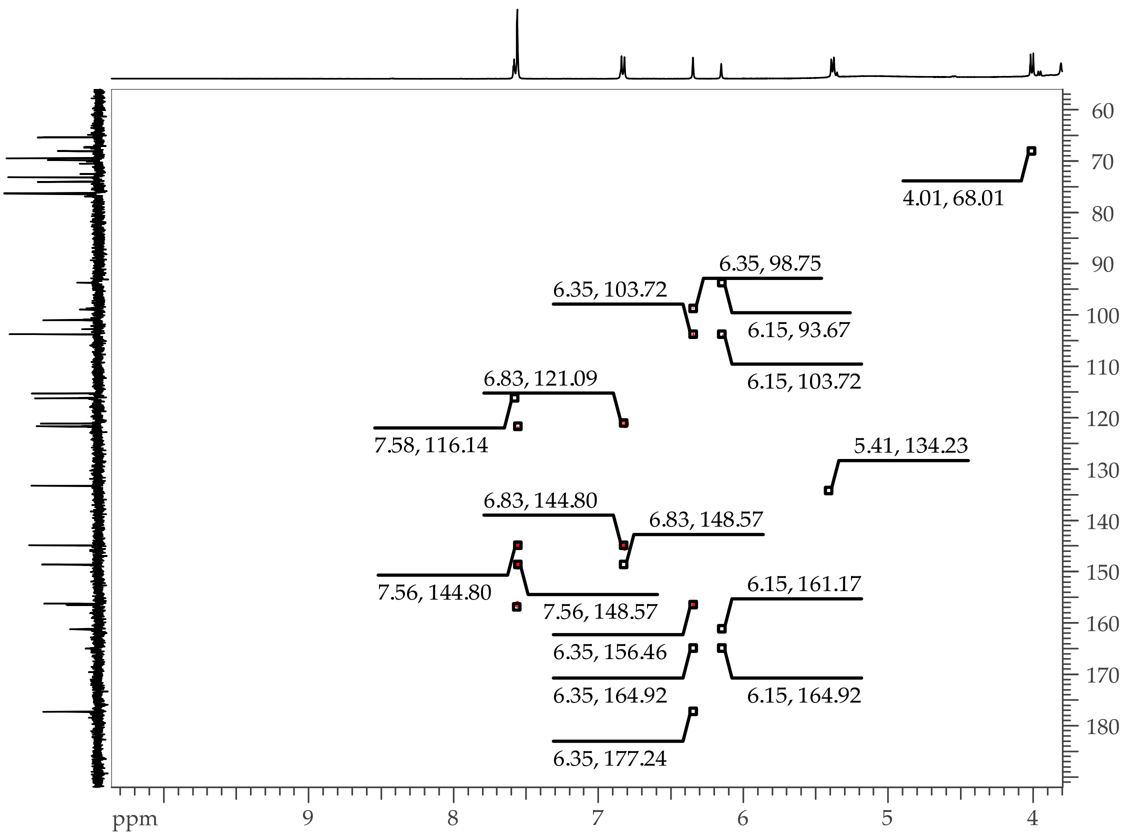

Figure S35: The HMBC spectrum of compound **18** in DMSO-*d6*.

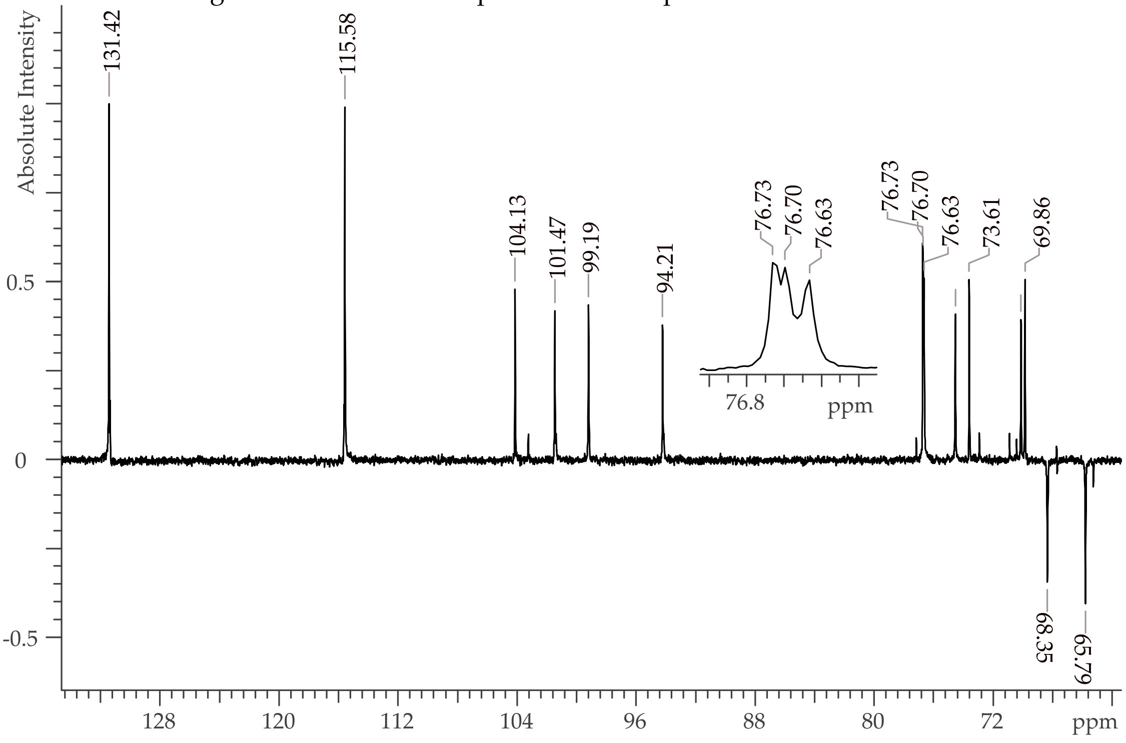

Figure S36: The DEPT spectrum of compound **16** in DMSO-*d6*.

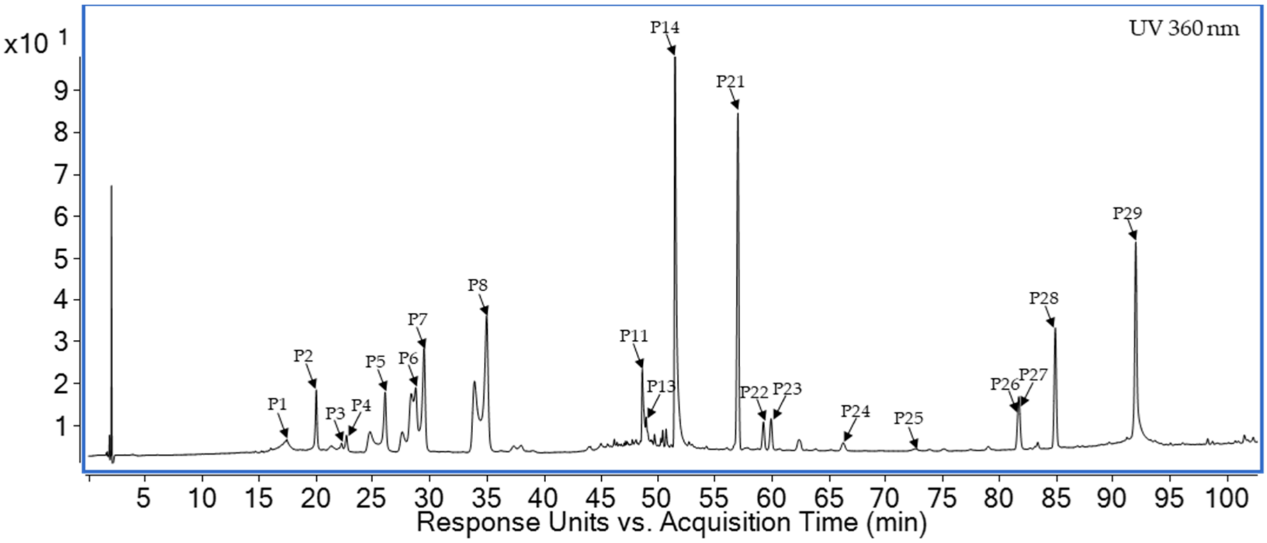

Figure S37: The UV chromatogram of extract **HP1** obtained by LC-PDA-HRMS.

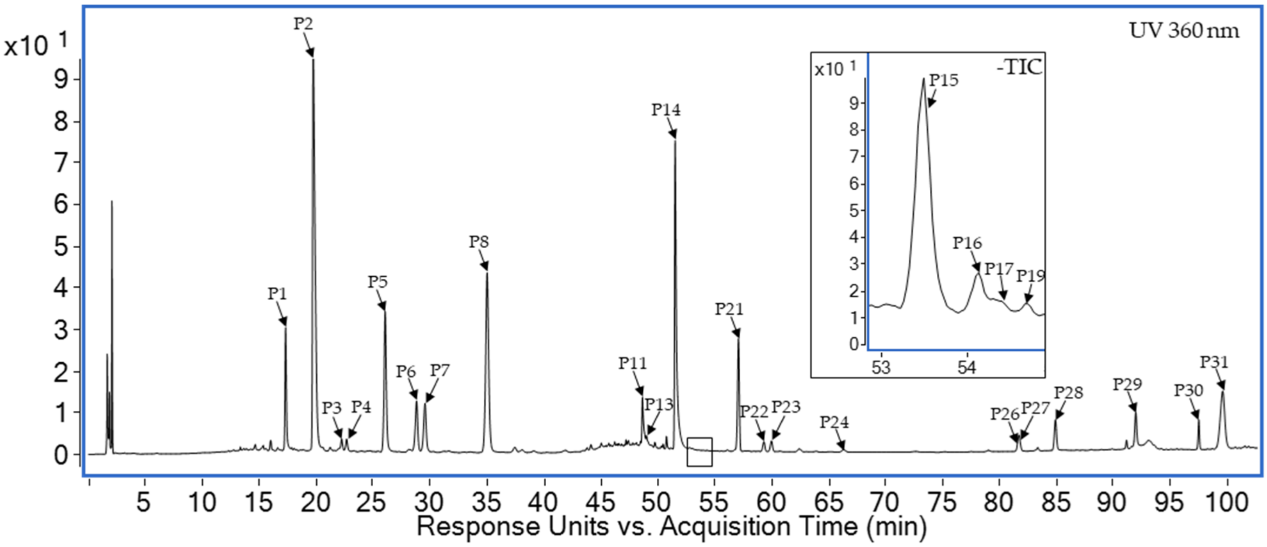

Figure S38: The UV with crucial MS chromatogram of extract **HP2** obtained by LC-PDA-HRMS.

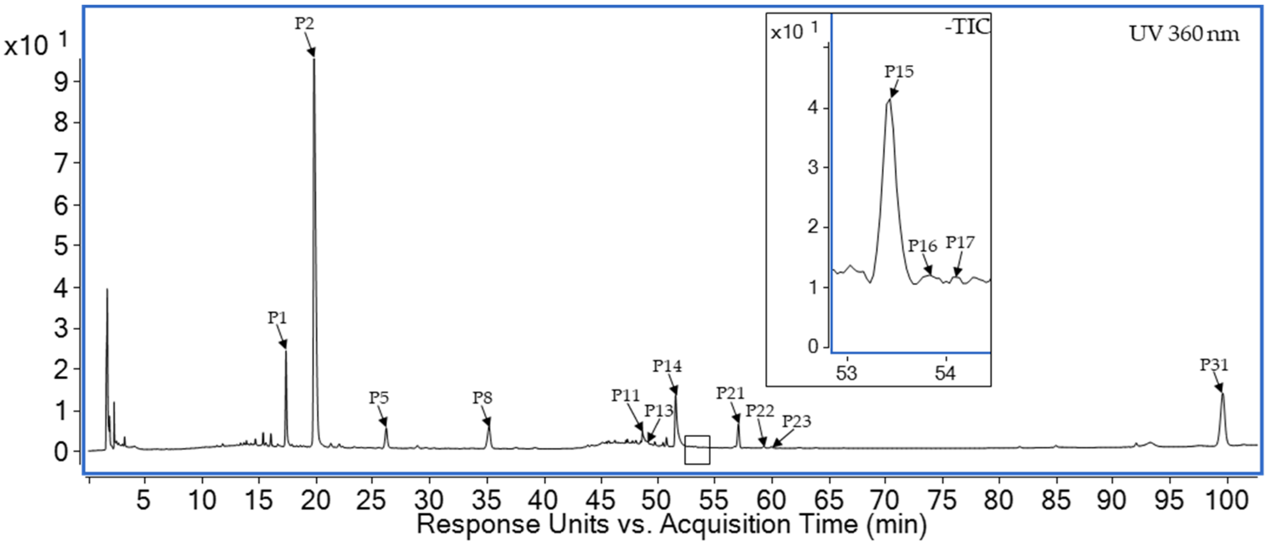

Figure S39: The UV with crucial MS chromatogram of extract **HP3** obtained by LC-PDA-HRMS.

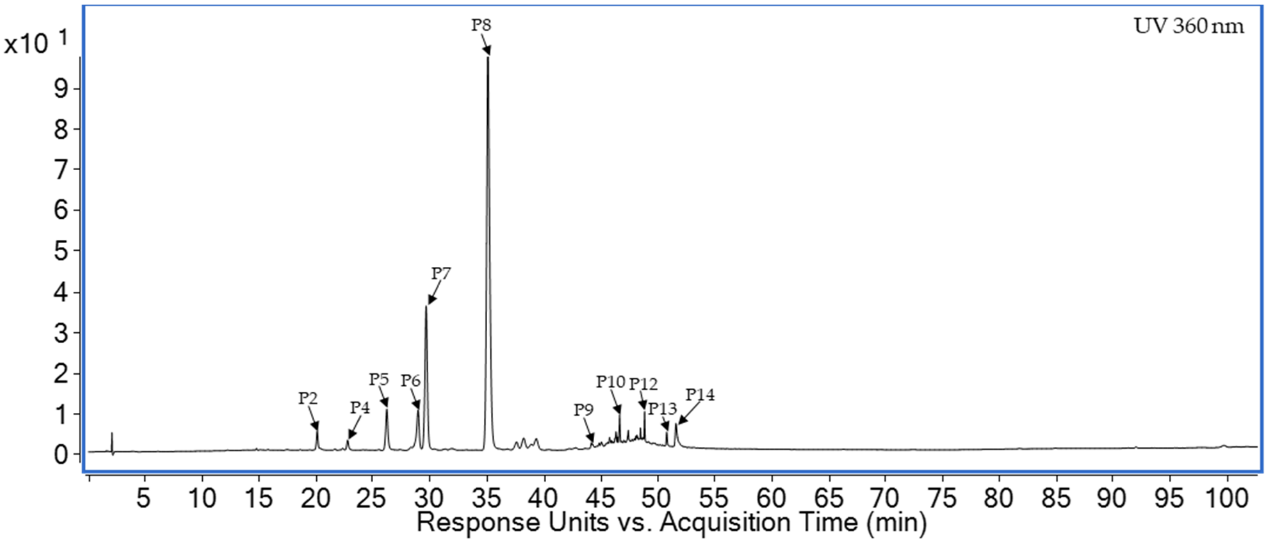

Figure S40: The UV chromatogram of fraction **HP4** obtained by LC-PDA-HRMS.

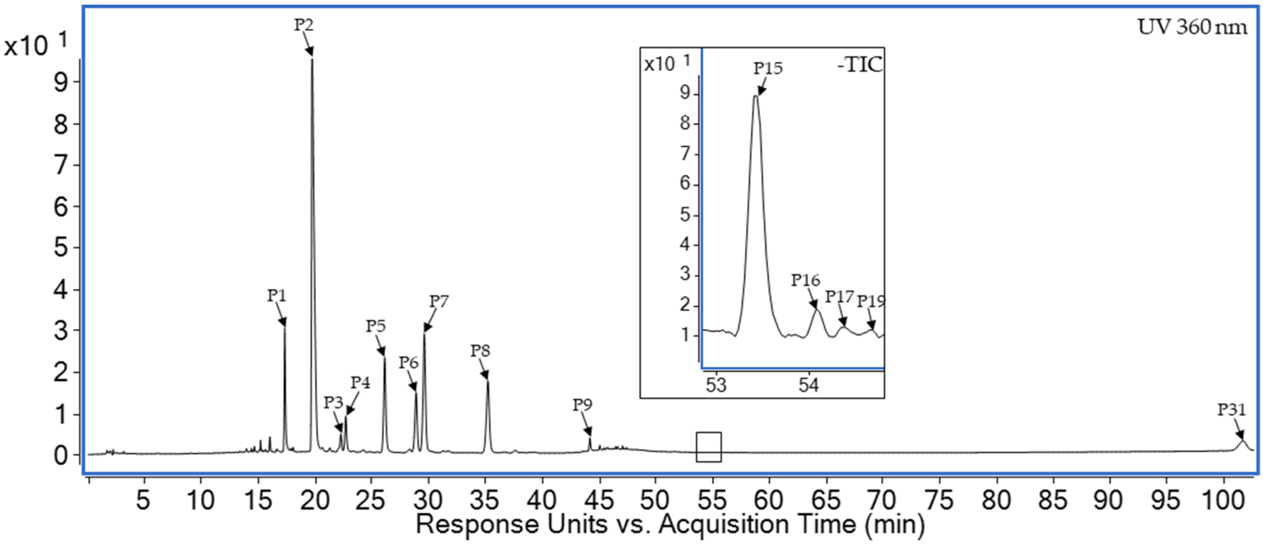

Figure S41: The UV with crucial MS chromatograms of fraction **HP5** obtained by LC-PDA-HRMS.

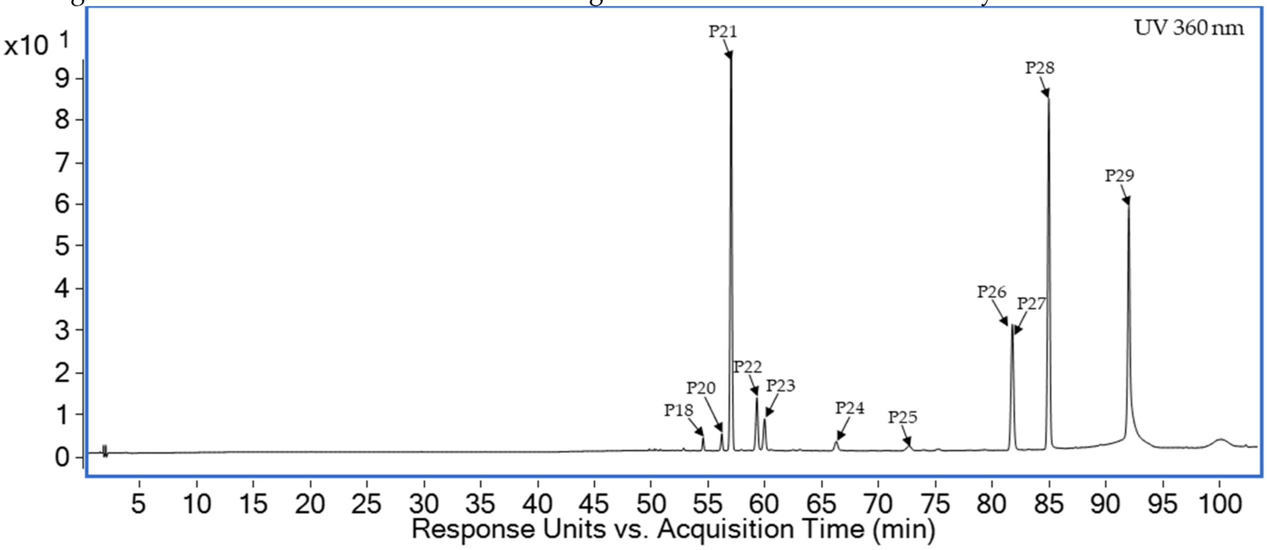

Figure S42: The UV chromatogram of extract **HP6** obtained by LC-PDA-HRMS.

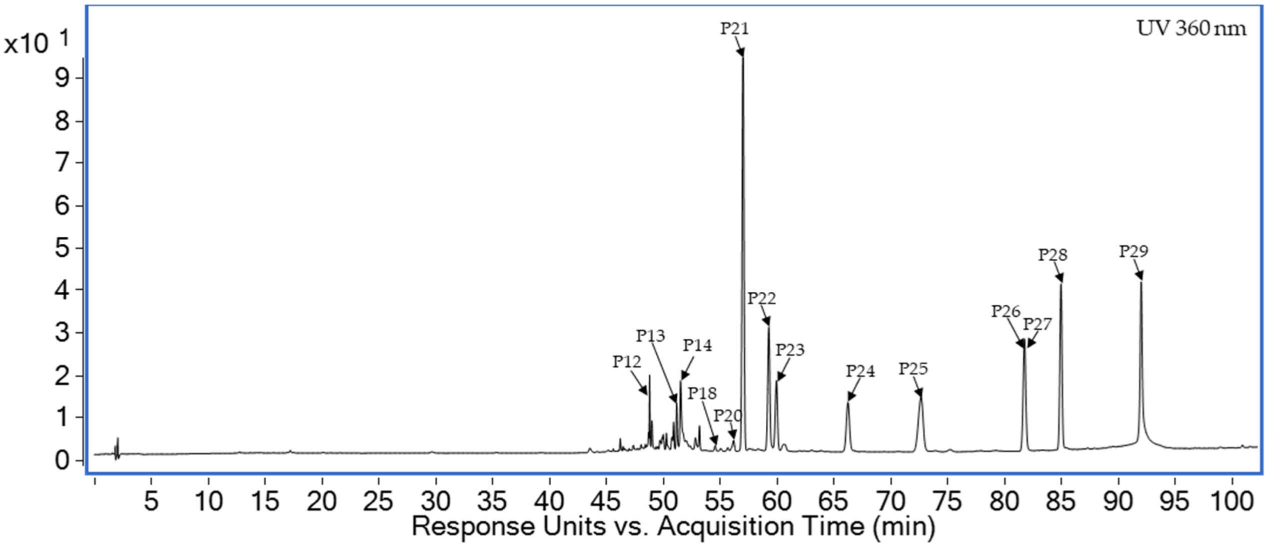

Figure S43: The UV chromatogram of extract **HP7** obtained by LC-PDA-HRMS.

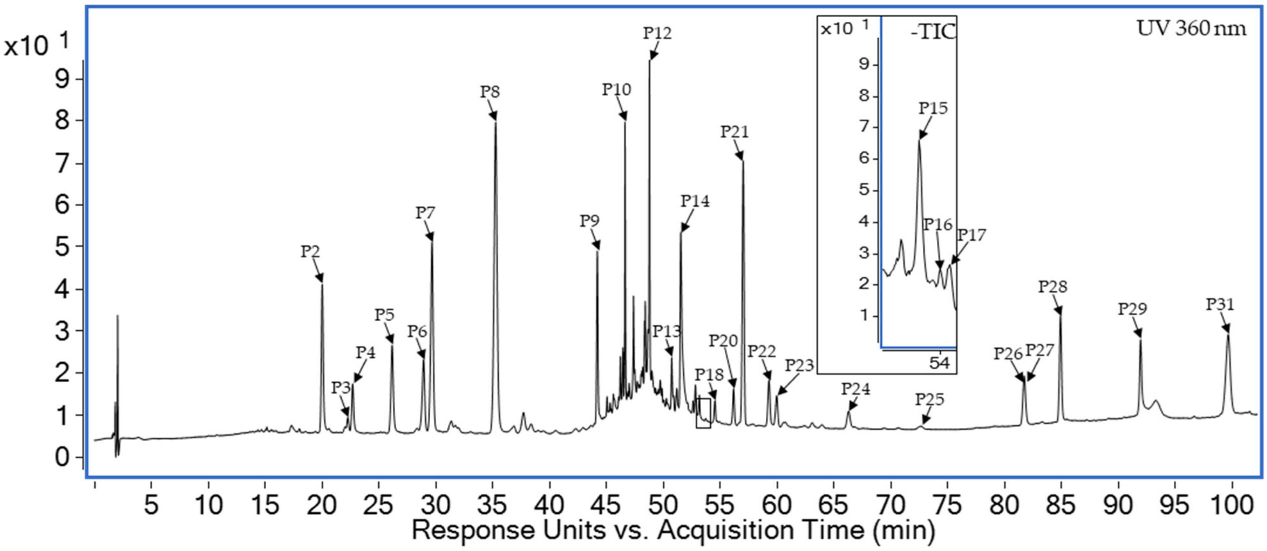

Figure S44: The UV with crucial MS chromatograms of fraction **HP8** obtained by LC-PDA-HRMS.
